# EucaMOD: a comprehensive multi‐omics database for functional genomics research and molecular breeding of fast‐growing eucalyptus trees

**DOI:** 10.1111/tpj.70603

**Published:** 2025-12-15

**Authors:** Meng Li, Yunpeng Cao, Wenfei Wu, Yi Mo, Jianzhong Wang, Xianchen Geng, Jiajing Xu, Yuchong Fei, Guofen Su, Hao Hu, Kuipeng Li, Jun Ni, Zeng‐Fu Xu

**Affiliations:** ^1^ Guangxi Key Laboratory of Forest Ecology and Conservation, State Key Laboratory for Conservation and Utilization of Subtropical Agro‐Bioresources, College of Forestry Guangxi University Nanning 530004 China; ^2^ Guangxi Colleges and Universities Key Laboratory for Cultivation and Utilization of Subtropical Forest Plantation, Key Laboratory of National Forestry and Grassland Administration on Cultivation of Fast‐Growing Timber in Central South China, College of Forestry Guangxi University Nanning 530004 China; ^3^ Guangxi Dongmen Forest Farm Chongzuo 532108 China

**Keywords:** epigenetics, eucalypt, genome, pan‐proteome, transcriptomics, woody plants

## Abstract

Eucalyptus, one of the most widely planted plantation tree species globally, is primarily found in tropical and subtropical regions and contributes significantly to economic and social benefits. With advances in sequencing technologies, there is an increasing demand for the systematic analysis of multi‐omics data among *Eucalyptus* species to enhance genetic breeding efforts. Although several early genomic databases have been established for eucalyptus, they have not been updated in a timely manner and lack recent multi‐omics data, rendering them insufficient for current research needs. To address this gap, we developed the eucalyptus multi‐omics database (EucaMOD, http://eucalyptusggd.net/eucamod), a comprehensive resource for cross‐omics studies. In this study, we functionally annotated 45 eucalyptus genomes and structurally annotated 15, conducting comparative genomics and pan‐proteomics analyses across all genomes. Additionally, we analyzed eucalyptus transcriptome, epigenome, and variome data through standardized workflows, enabling the in‐depth mining and reanalysis of multi‐omics datasets. EucaMOD is the most comprehensive multi‐omics database for eucalyptus to date and includes data from 45 genomes (39 species), 870 mRNA‐seq samples, 17 miRNA‐seq samples, 52 epigenomic datasets (histone modifications and transcription factor binding), and genetic variation data from 1219 samples. To support functional genomics and molecular breeding research, the database is organized into the following 11 modules: Home, Species, Genomics, Comparative genomics, Pan‐proteomics, Transcriptomics, Epigenetics, Variomics, Tools, Download, and Help. EucaMOD also offers online analysis tools for data mining, providing free public services to aid eucalyptus gene function and genetic engineering studies.

## Significance Statement

As eucalyptus research increasingly relies on big data, scientists have lacked a modern, integrated platform for multi‐omics analysis. We present a comprehensive eucalyptus multi‐omics database and analysis platform (EucaMOD) that unifies genomic, transcriptomic, and epigenomic data for 39 species, providing a vital resource to accelerate gene discovery and genetic improvement in eucalyptus.

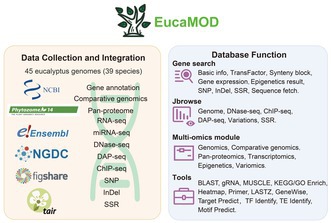

## INTRODUCTION

Eucalyptus, which encompasses species from the genera *Eucalyptus*, *Angophora*, and *Corymbia* in the Myrtaceae family, is native to Australia, predominantly distributed in tropical and subtropical regions, and ranks among the world's top three fast‐growing plantation tree species (Lee et al., 2024). As a key plantation species, eucalyptus is cultivated in 95 countries globally, with its total planted area surpassing 22.57 million hectares. Eucalyptus is renowned for its rapid growth, adaptability to diverse environments, and significant economic value, making it a cornerstone of forestry, paper, and woodworking products (Lee et al., 2022). Eucalyptus is a rich source of phytochemicals, including flavonoids, alkaloids, tannins, and phenylpropanoids, which are distributed throughout its leaves, stems, and roots. Numerous studies have evaluated the therapeutic potential of compounds derived from eucalyptus and demonstrated their beneficial properties, such as analgesic activity, antiviral action, anti‐inflammatory effects, antibacterial properties, antidiabetic benefits, antioxidative capacity, antitumor activity, antihistaminic effects, anticancer potential, and hepatoprotective effects, possessing various therapeutic effects (Shiekh et al., 2025). Given the economic, ecological, and medicinal significance of eucalyptus and its rapid growth and adaptability, the construction of a comprehensive multi‐omics database (eucalyptus multi‐omics database, EucaMOD) is essential to advancing functional genomics and molecular breeding efforts. This database will facilitate the identification of key genes and co‐expression networks underlying these traits and accelerate the development of superior eucalyptus varieties through advanced molecular breeding strategies.

The genetic improvement of eucalyptus is fundamentally shaped by its distinctive reproductive biology. Eucalyptus are predominantly outcrossing, but a mixed mating system is prevalent, characterized by a substantial incidence of self‐fertilization alongside cross‐pollination. This mating strategy preserves a high degree of heterozygosity and abundant genetic diversity within populations, providing indispensable genetic resources for breeding and selection programs (Potts, 2004; Potts et al., 2008; Ramalho et al., 2022). However, selfing precipitates pronounced inbreeding depression, diminishing progeny survival and growth vigor, which complicates genetic selection in open‐pollinated populations. Conventional eucalyptus breeding methodologies have evolved from initial provenance and population selection to the targeted development of superior families through controlled pollination, the exploitation of heterosis via interspecific hybridization, and the mass propagation and deployment of elite genotypes through clonal technologies—approaches designed to harness both additive and non‐additive genetic effects to maximize genetic gain (Eldridge, 1978; Ramalho et al., 2022). Despite substantial progress, traditional breeding strategies remain constrained by the lengthy generation times intrinsic to eucalyptus and the requirements for extended field phenotyping of economically important, late‐developing traits, such as wood yield and quality. These factors collectively impose fundamental limits on the rate of genetic advancement achievable per unit time (Chauhan et al., 2014; Wang, Wu, et al., 2024).

To overcome the bottlenecks inherent to conventional breeding and expedite genetic improvement, contemporary eucalyptus breeding has shifted toward strategies that utilize molecular technologies. Early efforts employing marker‐assisted selection (MAS) sought to leverage a limited number of major quantitative trait loci (QTLs) tightly linked to target traits. However, since key economic traits in eucalyptus—including the growth rate and wood properties—are quantitatively inherited and governed by numerous minor‐effect genes, the practical utility of MAS has proven extremely limited. This challenge has catalyzed a paradigm transition to genomic selection (GS) (Lebedev et al., 2020). GS does not require the identification of individual QTLs, instead utilizing genome‐wide, high‐density molecular markers to construct predictive models for estimating genomic breeding values (Simiqueli et al., 2023). By enabling precise early selection at the seedling stage, GS shortens the breeding cycle, enhances the selection intensity and accuracy, and can more than double the rate of genetic gain per unit time (Duarte et al., 2024; Resende et al., 2017). Consequently, GS is currently regarded as the most promising frontier in eucalyptus genetic improvement. The transition to GS‐centric breeding highlights the pivotal importance of constructing comprehensive genomic resources; the extensive, high‐quality multi‐omics and molecular marker data embedded in EucaMOD constitute an indispensable foundation for implementing advanced molecular breeding strategies and accelerating the development of improved eucalyptus cultivars.

The genomics era has significantly advanced our understanding of eucalyptus biology. In 2014, the *Eucalyptus grandis* genome was sequenced, providing a foundational resource for genetic research and breeding programs (Myburg et al., 2014). In 2023, the high‐quality 545.75 Mb *E*. *urophylla* × *E*. *grandis* hybrid genome was assembled, with 34 502 protein‐coding genes annotated (Shen et al., 2023). Researchers successfully assembled high‐quality haploid genomes for *E*. *grandis* and *E*. *urophylla*, achieving 98.0% BUSCO completeness with genome sizes of 566.7 and 544.5 Mb, respectively, and scaffold N50s of 43.8 and 42.5 Mb, respectively (Lotter et al., 2022). Since then, the genomes of several other economically important species, including *E*. *camaldulensis*, *E*. *cloeziana*, *E*. *globulus*, and other *Eucalyptus* species (30 *Eucalyptus*, 1 *Angophora*, and 1 *Corymbia*), have been assembled and annotated (Ferguson, Jones, et al., 2024; Li, Wu, et al., 2025). A high‐quality, near telomere‐to‐telomere (T2T) complete, haplotype‐resolved diploid genome reference for *E*. *regnans* has been assembled, revealing extensive structural variations and gene content differences between haplotypes (Ferguson, Bar‐Ness, et al., 2024). The integration of multi‐omics data, including genomics, transcriptomics, and epigenomics, has further deepened our understanding of eucalyptus biology and its molecular mechanisms. These advancements have laid the foundation for accelerating molecular breeding efforts and improving the sustainability of eucalyptus‐based industries.

To better utilize genomics data, genomics analysis platforms have been developed for eucalyptus. For instance, the EucaMaps database (https://arachne.pierroton.inrae.fr/eucamaps/index.html) integrates 267 QTLs for five traits in six *Eucalyptus* species and one *Corymbia* species with 11 linkage groups into an online analysis platform (Gion et al., 2014). The EucGenIE database (https://eucgenie.org/) integrates genome and transcriptome data for *E*. *grandis* (Myburg et al., 2014). The EUCANEXT database (http://bioinfo03.ibi.unicamp.br/eucalyptusdb/) contains genome sequences for *E*. *grandis* and *E*. *camaldulensis*, transcriptome data for five *Eucalyptus* species and Expressed Sequence Tag (EST) data for six *Eucalyptus* species (Nascimento et al., 2017). Nevertheless, these early established eucalyptus databases, which are limited in data and lack recent multi‐omics updates and integration, cannot meet modern research needs. Therefore, we developed EucaMOD, a free, accessible, and user‐friendly platform. EucaMOD integrates all currently available eucalyptus genomes, including the most recent haploid genome of *E*. *grandis* × *E*. *urophylla* and the T2T genome of *E*. *regnans* (Ferguson, Bar‐Ness, et al., 2024; Lotter et al., 2022), offering researchers a robust platform for advancing functional genomics and molecular breeding research in eucalyptus.

## RESULTS AND DISCUSSION

### Database overview

**Figure 1:**
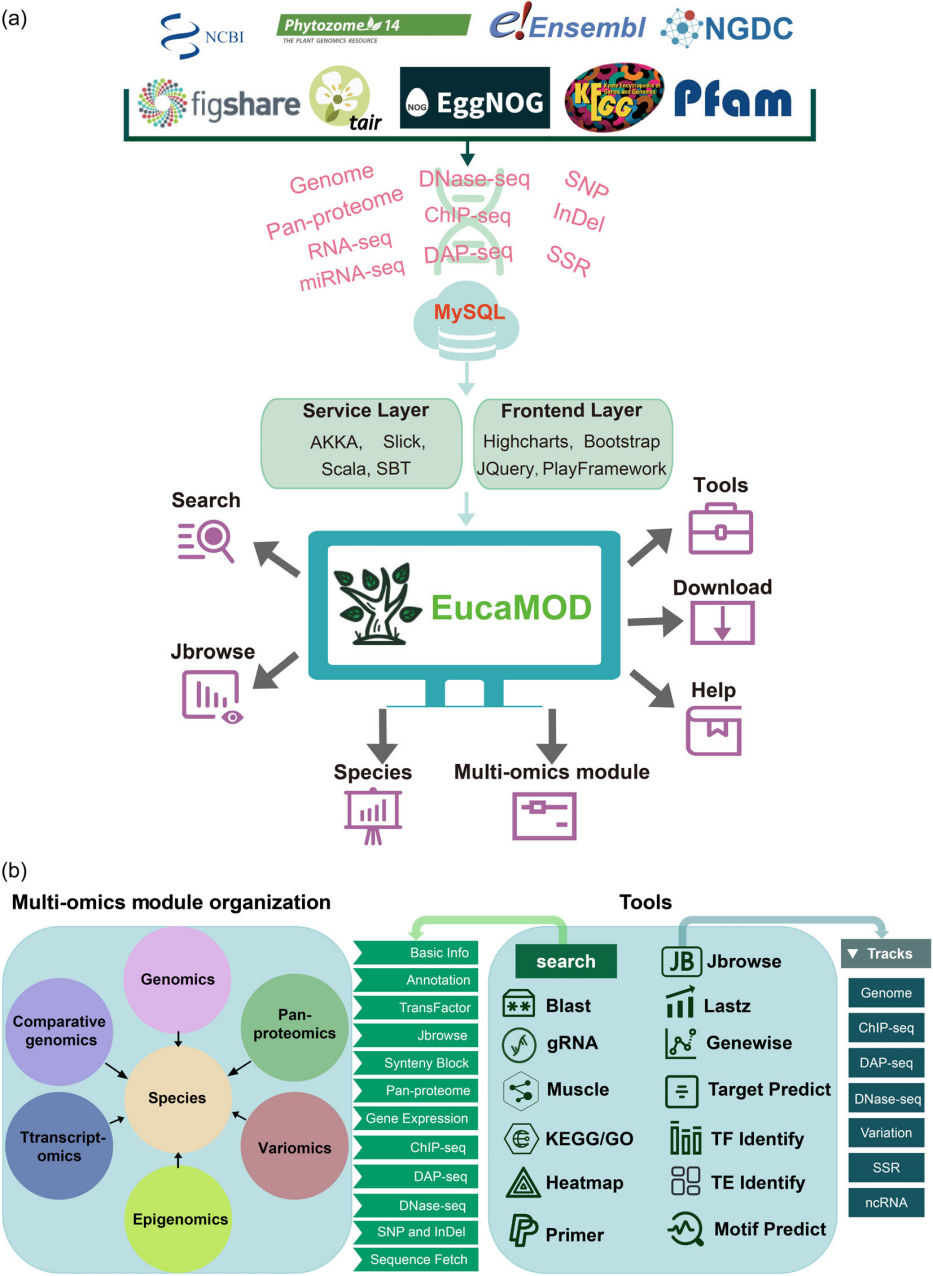
Construction pipelines and overview of EucaMOD. (a) Software architecture and data integration pipeline of EucaMOD. (b) Module organization and available tools in EucaMOD.

Based on the analysis results from diverse omics datasets, we developed and integrated a variety of tools for data querying, browsing, analysis, and visualization, constructing EucaMOD (http://eucalyptusggd.net/eucamod). As shown in Figure 1a, the platform integrates multi‐omics data from public databases and in‐house datasets into a MySQL‐based backend, supported by a service layer (AKKA, Slick, Scala, and SBT) and a front‐end layer (Highcharts, Bootstrap, JQuery, and PlayFramework). This architecture enables seamless connections among data storage, search functions, interactive browsers, and analytical tools. EucaMOD encompasses the following 11 modules: Home, Species, Genomics, Comparative genomics, Pan‐ proteomics, Transcriptomics, Epigenetics, Variomics, Tools, Download, and Help (Figure 1b). The statistical results of the database are presented on the homepage. The platform's homepage features a powerful multi‐omics search tool at the top right, enabling users to quickly search the database by entering the gene ID, name, or symbol of interest to retrieve relevant multi‐omics information, including gene location, gene annotation, transcription factor (TF) identification, JBrowse tracks, gene expression, visualization of comparative genomics, pan‐proteomics, epigenomics, variomics, and sequence fetch of transcripts, coding sequences (CDSs), and proteins (Figure S1). EucaMOD is a comprehensive multi‐omics platform for eucalyptus research that offers users a one‐stop solution for data acquisition and online analysis.

### Species module

The Species module was intended to provide germplasm data to support research on tree breeding and domestication and was designed to provide comprehensive species‐level data for the 39 *Eucalyptus* species included in EucaMOD. Each species has a dedicated page. Taking *E. urophylla* as an example (Figure S2), researchers can access detailed descriptive information for a more thorough species assessment. The description consists of the following parts: passport data (including species, genus, family, description, taxonomy ID, NCBI link, and WFO link); morphological images (such as leaf, flower, and fruit); and genomic information for all genotypes of the species in the database (including genome size, assembly level, assembly version, and source). In addition, the species name is hyperlinked to its genome page, where users can browse gene information. To facilitate interactive exploration, the species page incorporates a comparative genomics panel, enabling users to examine synteny relationships between the selected species and other *Eucalyptus* species genomes. This interactive design enhances data accessibility and empowers researchers to intuitively identify conserved genomic regions and species‐specific variations, accelerating comparative genomic studies in eucalyptus.

### Genomics module

**Figure 2:**
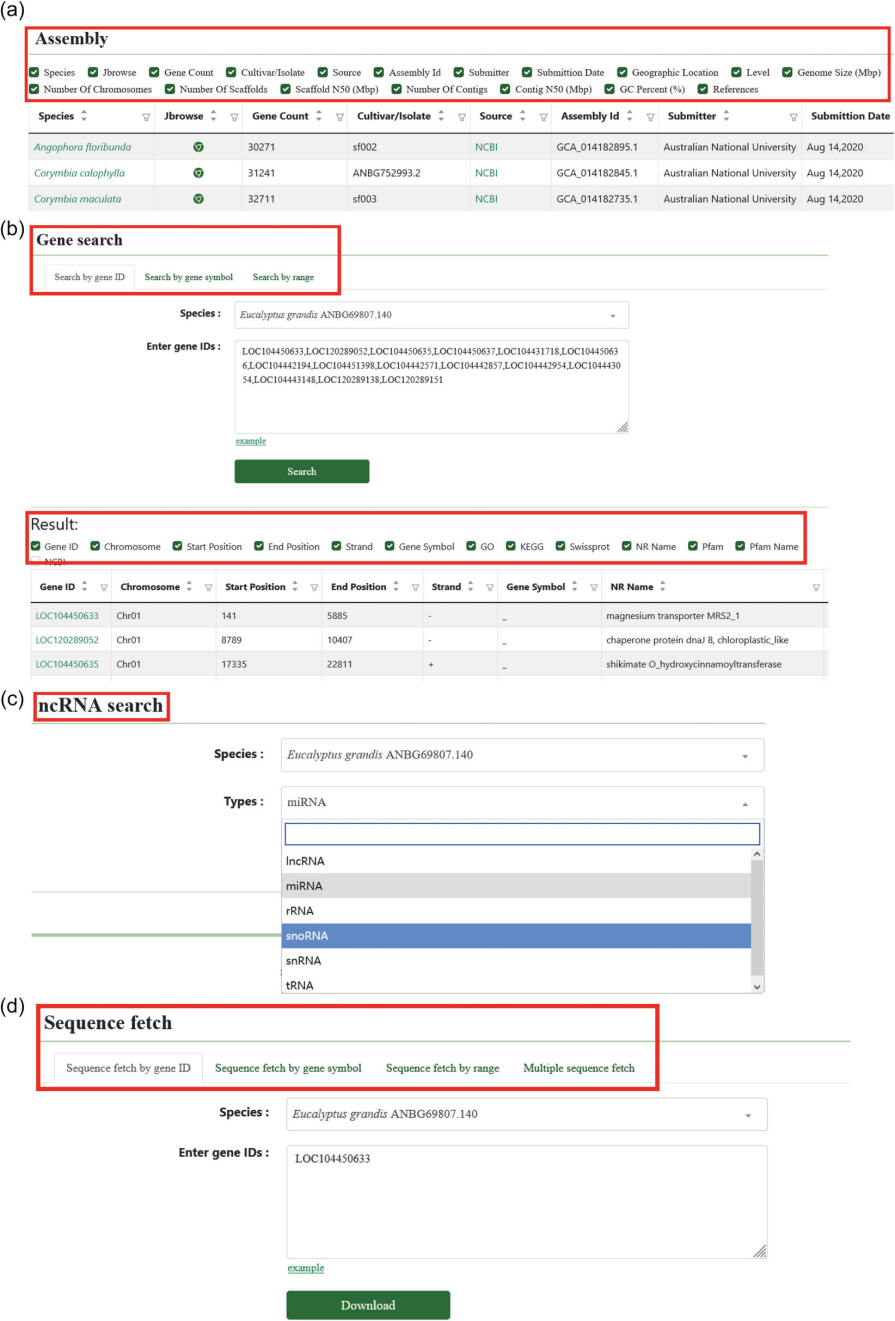
Framework of the genomics module in EucaMOD. (a) Genome assembly information can be viewed for all genomes in the Assembly submenu. (b) Gene search and results page. (c) ncRNA search and results page. (d) Gene sequences can be downloaded from the Sequence fetch submenu.

The Genomics module includes submenus for Genome assembly, Gene search, ncRNA search, and Sequence fetch, integrating genome assembly data from multiple public databases, encompassing 45 genomes, namely *Angophora floribunda*, *C*. *calophylla*, and *C*. *maculata* genomes and 42 genomes from the *Eucalyptus* genus (Figure 2a, Table S1) (Ferguson, Bar‐Ness, et al., 2024; Ferguson, Jones, et al., 2024; Lotter et al., 2023; Shen et al., 2023). For each genome, users can access basic information, such as the species name, genome size, assembly level, assembly version, source, number of scaffolds, N50 value, and GC content. The gene search and non‐coding RNA (ncRNA) search functionalities allow users to query target genes and ncRNAs by gene ID, gene name, or chromosomal location, providing annotations, such as gene position, Gene Ontology (GO) and Kyoto Encyclopedia of Genes and Genomes (KEGG) pathways, Swiss‐Prot, NR, and Pfam database annotation information (Figure 2b,c, Table S2), which users can query and download for their target genes. The gene is linked to multi‐omics data and can be viewed in JBrowse. The 15 genomes are re‐annotated in Table S3. Sequence fetch enables users to obtain sequences by gene ID, gene name, or chromosomal location (Figure 2d).

### Comparative genomics module

**Figure 3:**
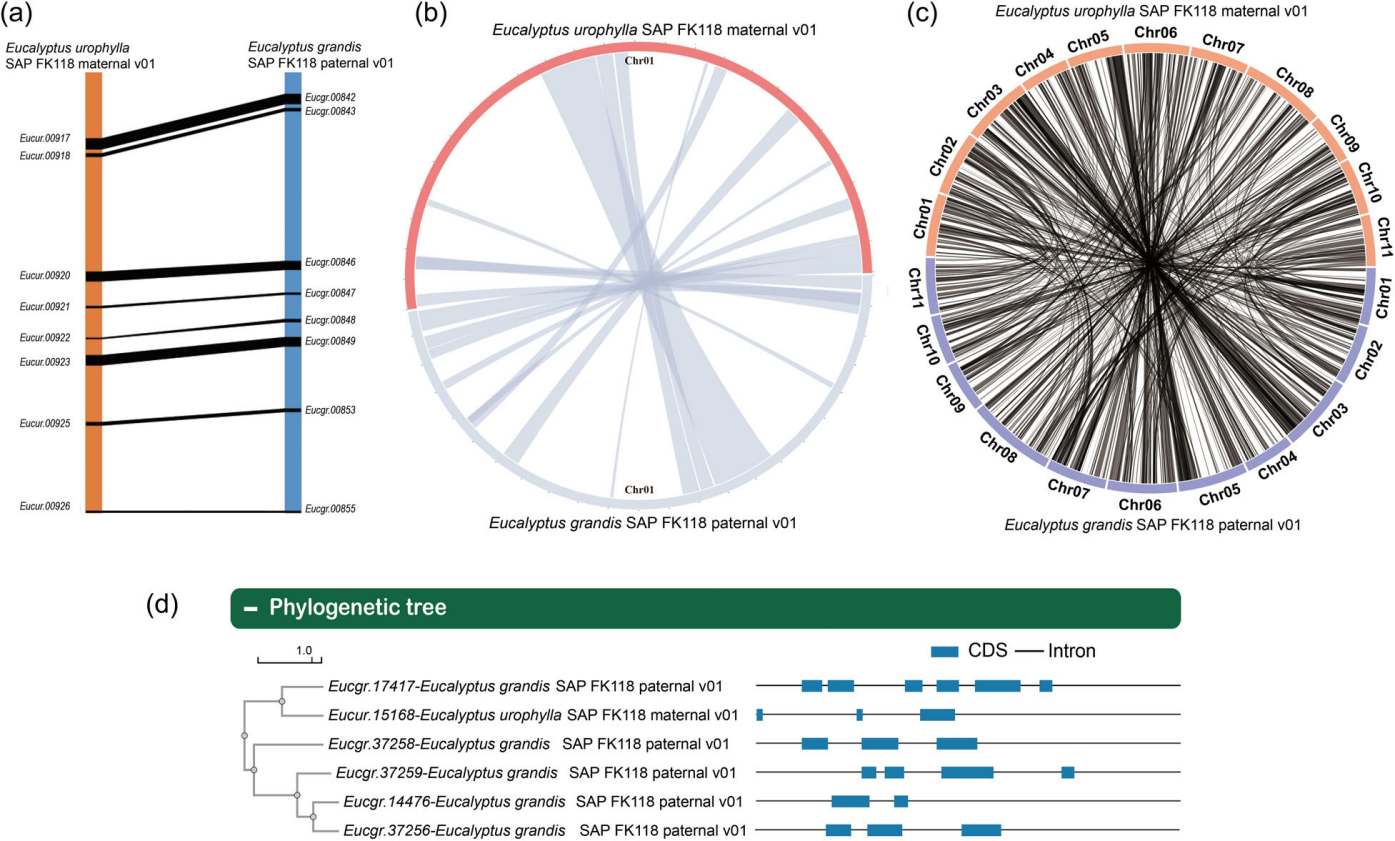
Functions provided by the comparative genomics module. (a) GenePair interface displaying collinear gene blocks between *Eucalyptus urophylla* SAP FK118 and *E. grandis* SAP FK118. (b) MicroCollinearity visualization of chromosome‐level syntenic regions between chromosome 1 (Chr01) of *E*. *urophylla* SAP FK118 and *E. grandis* SAP FK118. (c) Macrocollinearity overview showing whole‐genome alignment blocks between *E*. *urophylla* SAP FK118 and *E*. *grandis* SAP FK118. (d) Gene phylogenetic tree in the Homologs submenu.

To systematically investigate the genomic diversity and evolutionary dynamics among *Eucalyptus* species, we conducted comparative genomics analysis of 45 eucalyptus genomes, which were available in the Comparative genomics module. Based on single‐copy gene families, a phylogenetic tree was constructed to illustrate the evolutionary relationships among *Eucalyptus* species (Figure S3). The GenePair, MicroCollinearity, and MacroCollinearity tools can be used to query orthologous gene pairs and analyze collinearity at the gene, chromosome, and genome levels (Figure 3a–c). Additionally, the Homologs submenu provides access to orthologs and paralogs, as well as the phylogenetic tree and gene structure visualization of homologous genes, assisting users in identifying key genes (Figure 3d). The design of this module holds significant value for researchers, as it facilitates the exploration of evolutionary relationships and functional divergence among *Eucalyptus* species. By integrating collinearity analysis and phylogenetic tools, the comprehensive approach enhances our understanding of eucalyptus genomics and provides a robust foundation for molecular breeding and genetic improvement studies.

### Pan‐proteomics module

**Figure 4:**
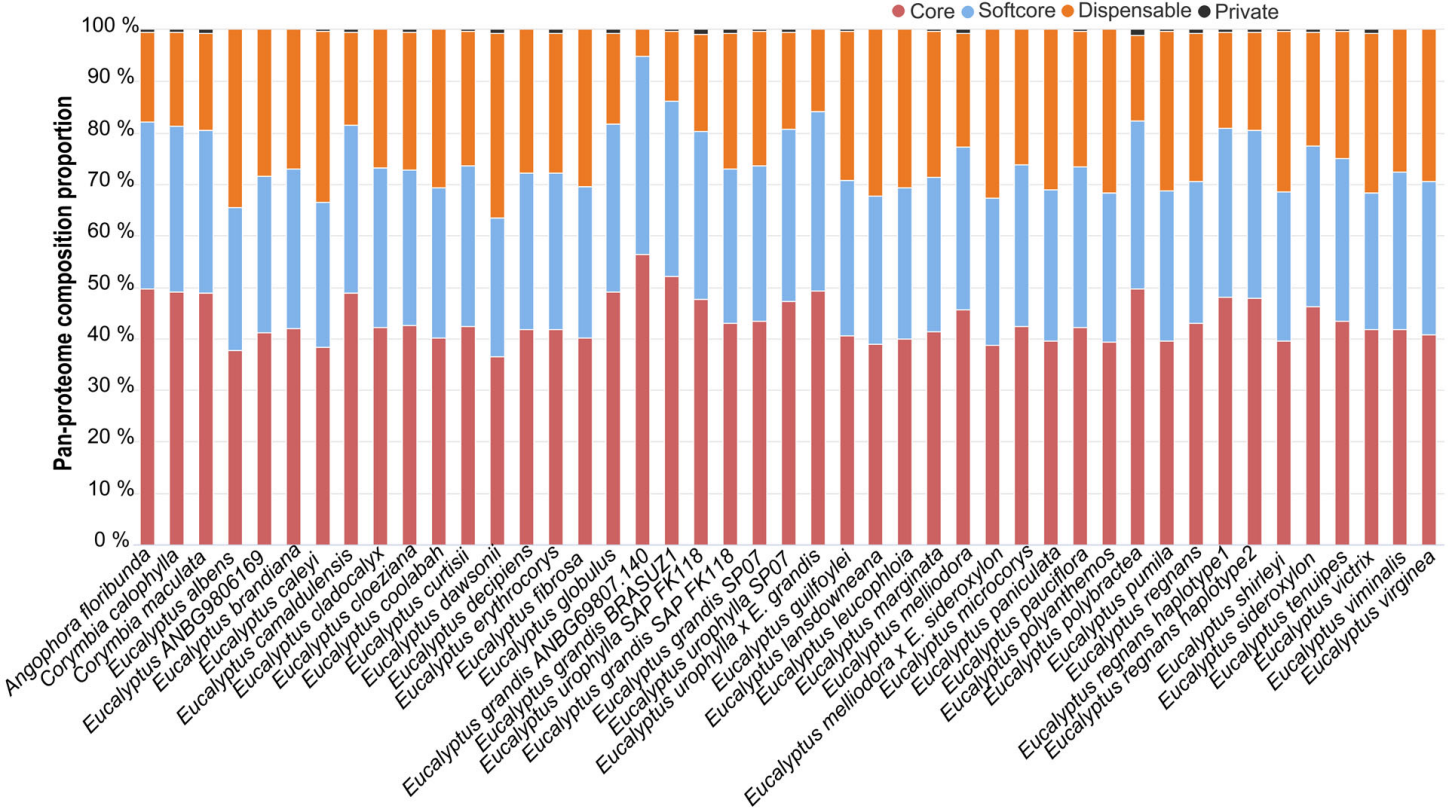
Pan‐proteome composition for each *Eucalyptus* species.

To explore the genomic diversity of *Eucalyptus* species and their dynamic changes during evolution, we developed a Pan‐proteomics module for *A*. *floribunda*, *C*. *calophylla*, *C*. *maculata*, and all *Eucalyptus* species. The genes from all genomes in EucaMOD were clustered into 50 875 gene families, composed of 8580 core (present in all genomes), 6425 softcore (present in 36–44 genomes), 33 552 dispensable (present in 2–35 genomes), and 2318 species‐specific gene families (Table S4). Users can query the proportion of each gene family in different species in the Pan‐proteome statistics submenu (Figure 4) and retrieve detailed gene information corresponding to core, softcore, dispensable, and species‐specific gene families for each *Eucalyptus* species through the Genes submenu.

Although *Angophora* and *Corymbia* species were classified in genera separate from *Eucalyptus*, they exhibited a distribution of gene family categories—core, softcore, dispensable, and species‐specific—analogous to that observed among *Eucalyptus* species. This suggests a conserved genomic architecture and shared evolutionary pressures across these related genera within the Myrtaceae family. The presence of a substantial dispensable genome fraction reflects ongoing genomic plasticity and may contribute to species‐specific adaptations and ecological diversification. Moreover, the core gene families likely represent essential functions conserved in all examined species that underlie the fundamental biological processes in this lineage. When compared to previous work on 33 *Eucalyptus* genomes, which reported that 21.33% (14552) of orthogroups were core, 76.00% (51858) were dispensable, and only 2.67% (1821) were private (species‐specific) (Ferguson, Jones, et al., 2024), our results showed both similarities and distinctions. In our expanded study (from three genera), the proportion of core gene families (8580) accounted for 16.86% of all families, which is slightly lower than the core fraction reported previously. This reduction likely reflects the broader phylogenetic scope of our study: genes considered core within a single genus may no longer meet the criteria for core status when multiple genera are analyzed, instead being reassigned to the softcore or dispensable categories. Both studies highlight that the majority of the pangenome is composed of dispensable genes, revealing their potential role as a major reservoir for adaptive traits and phenotypic diversity.

Similar patterns of pangenome composition have been observed in other plant families. In Solanaceae, a pangenome constructed from 30 representative genomes identified 43 395 syntenic gene families and classified them into high‐retention (>80% of species), medium‐retention (20–80%), and low‐retention (<20%) categories, revealing that many genes are variably present across species (Zhang et al., 2025). In the orange subfamily, analysis of 16 genomes showed that 35.8% of gene families were core and softcore, 55.4% were dispensable, and 8.8% were species‐specific, emphasizing the prevalence of non‐core genes (Huang et al., 2023). In legumes, a pangenome built from 12 genomes categorized 12 436 core, 1794 softcore, 14 223 shell, and 6936 cloud gene families, with core genes representing 41–70% of all genes in each species (Wang et al., 2025). Similarly, in our study, core genes also constitute the majority of the genes in the genome of each examined species (Figure 4). These cross‐family comparisons suggest that while the absolute proportions of core, variable, and species‐specific genes vary among lineages—likely due to differences in evolutionary history, genome duplication events, and ecological adaptation pressures—the general pattern of a substantial dispensable gene pool is a common feature in plant pangenomes. This dispensable fraction likely plays a key role in the adaptation to diverse environments and the emergence of lineage‐specific traits. Thus, our pan‐proteomics resource for Myrtaceae provides a valuable foundation for exploring these evolutionary dynamics and identifying candidate genes that may be leveraged in molecular breeding and genetic improvement of *Eucalyptus* and related species.

### Transcriptomics module

**Figure 5:**
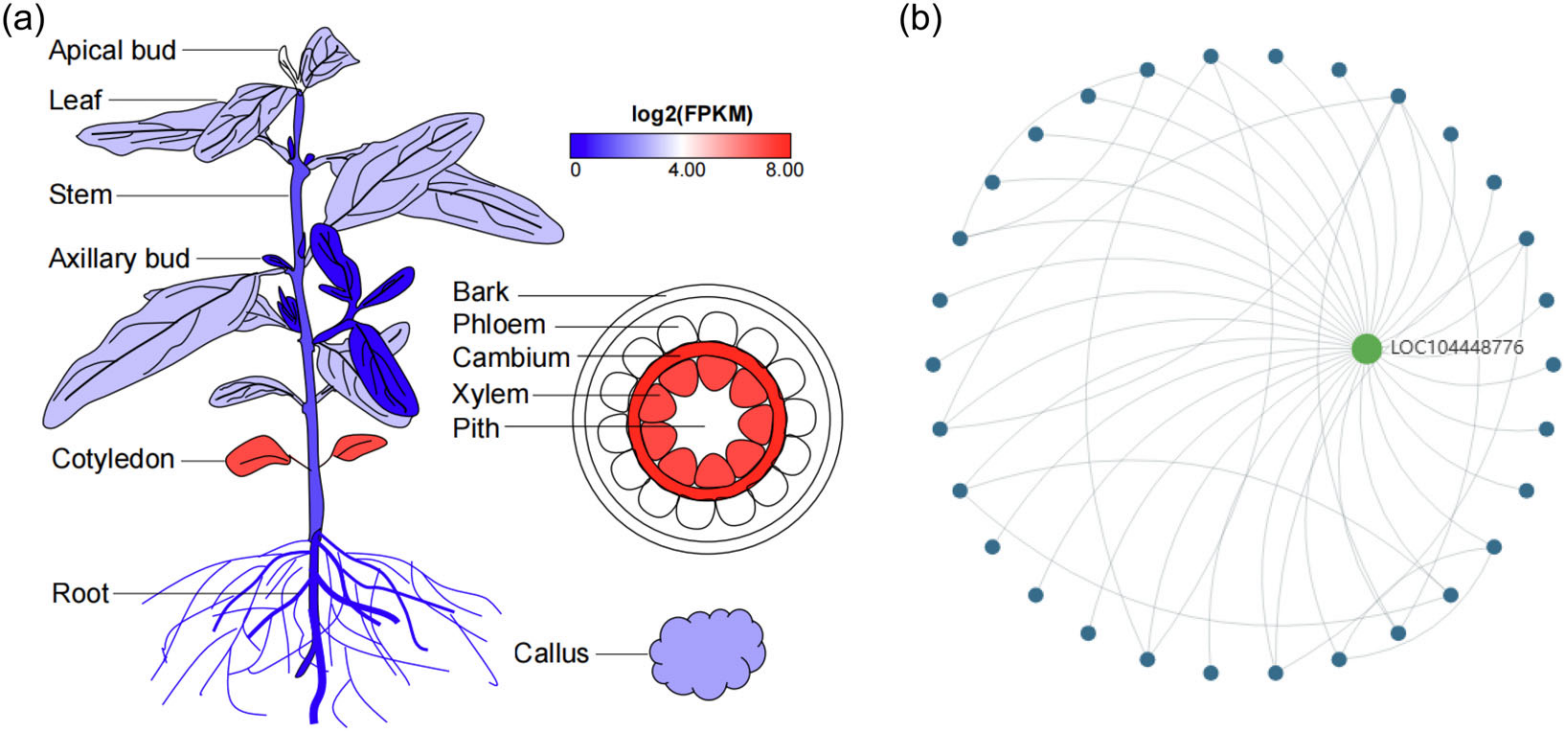
The transcriptomics module in EucaMOD. (a) Gene (EI154_mgp08) expression across different tissues, visualized using the eucalyptus eFP browser. (b) Gene (LOC104448776) co‐expression network map of *Eucalyptus grandis* in the Co‐expression network submenu.

The Transcriptomics module provides evidence of molecular mechanisms involving genes. To obtain gene expression data from a broader range of germplasm, we collected, analyzed, and integrated RNA‐seq data from 870 samples of 10 species and miRNA‐seq data from 17 samples of 3 species (Tables S5 and S6). In the RNA‐seq dataset, users can select a species to view sample information tables for different tissues and experimental conditions, enabling them to examine the expression patterns of genes of interest across all samples. To accommodate specific research interests, we added category labels in the RNA‐seq dataset and Gene expression profile submenus, enabling users to efficiently access the genes that meet their specific research interests. The module also supports the visualization and download of gene expression profiles among tissues for electronic fluorescent pictographs (eFPs) and other image types (Figure 5a). Users can download the expression and visualization results for lncRNA and miRNA through ncRNA expression (Figure S4). The transcriptomics module provides gene co‐expression network information and can be used to generate visual maps (Figure 5b). The transcriptomic data and tools can assist researchers in gaining a broader perspective of the functional and expression characteristics of genes of interest.

### Epigenomics module

**Figure 6:**
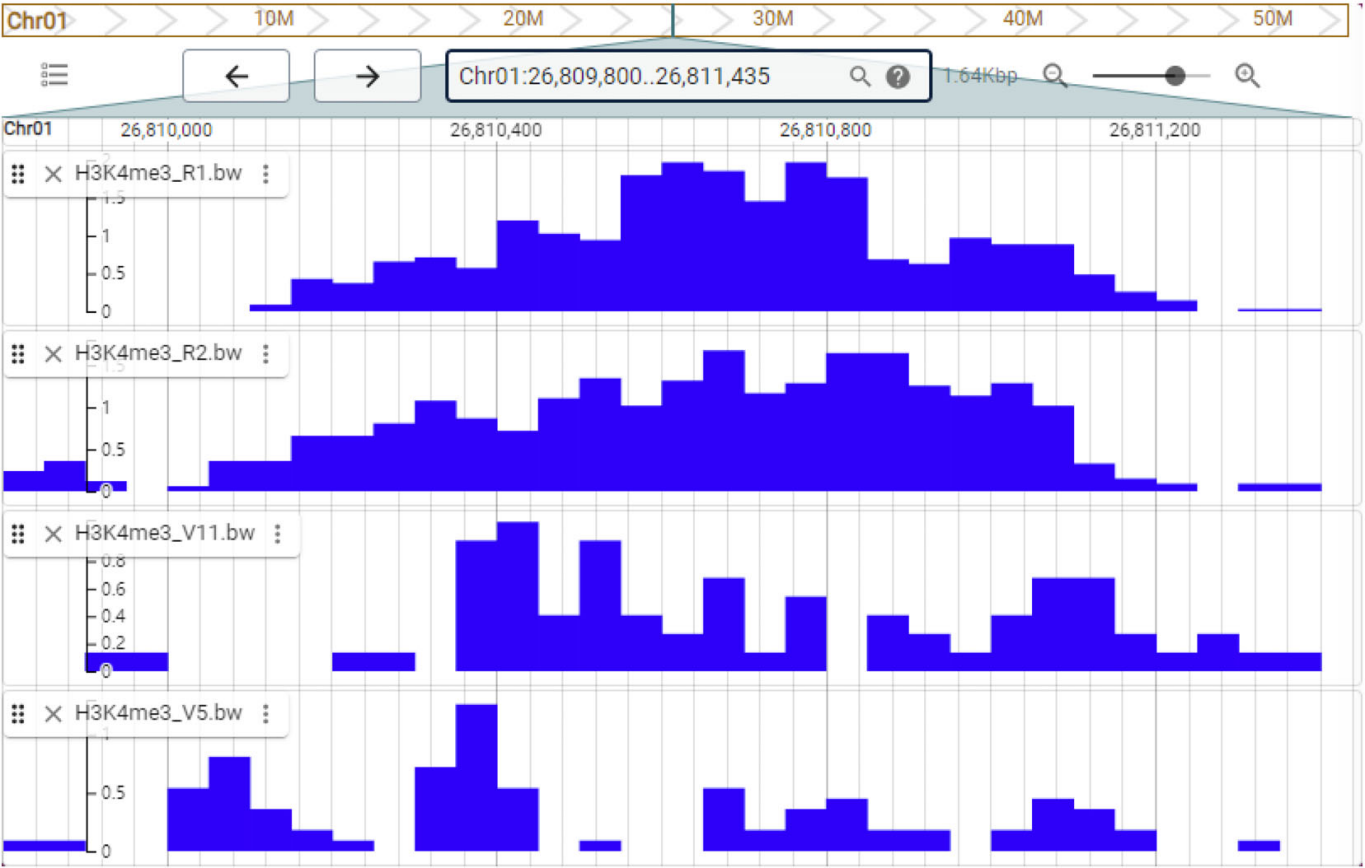
Visualization result page of the epigenomics module in EucaMOD. Visualization of H3K4me3 results among *Eucalyptus grandis* samples.

The Epigenomics module integrates comprehensive epigenetic data, including histone modifications (H3K4me3 and H3K27me3) and 10 TF binding sites (MYB1, MYB2, MYB20, MYB46, MYB69, MYB83, MYB85, MYB103, MYB122, and MYB135), which can be analyzed and integrated into the corresponding submenu (Table S7). These data provide valuable insights into the functional landscape of eucalyptus genomes, enabling researchers to evaluate the interplay between epigenetic modifications and gene expression. Users can query epigenetic data using the gene ID or chromosomal position within the corresponding epigenetics modules, facilitating targeted investigations into genomic regions or genes of interest (Figure 6). Additionally, all epigenetic data tracks for gene fragments can be visualized and explored using JBrowse, offering an interactive and user‐friendly interface for in‐depth analysis. EucaMOD provides the first systematic epigenetic data resource for eucalyptus that is helpful in identifying epigenetic regulatory elements and understanding their molecular mechanisms.

### Variomics module

**Figure 7:**
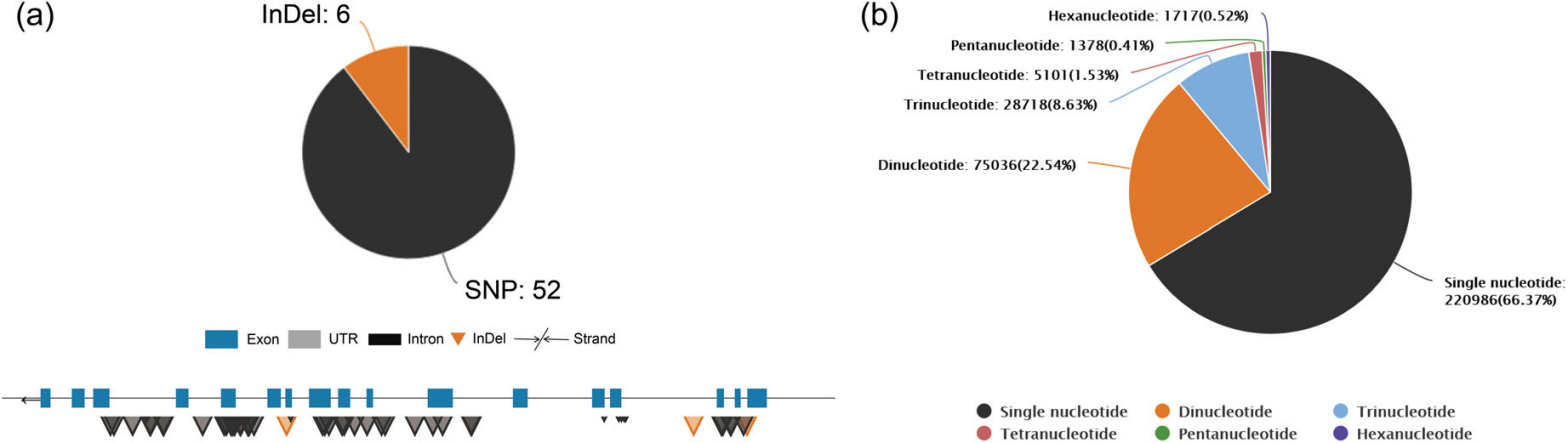
Visualization result page of the variomics module in EucaMOD. (a) Descriptions of genomic variation (SNPs and InDels) in the variomics module. (b) Visualization of SSR statistical results in *Eucalyptus grandis* ANBG69807.140.

Single nucleotide polymorphisms (SNPs) and insertions and deletions (InDels) for 11 *Eucalyptus* species that we analyzed and integrated (Table S8) can be browsed in the Variomics module. Users can search by gene ID to view the statistical results of variations, gene structures, and their positions (Figure 7a). Variations can also be explored in detail using JBrowse, which provides an interactive and user‐friendly interface for visualizing variomics data in the context of reference genomes. This feature enables researchers to investigate the distribution and potential functional impacts of genetic variations across the *E* *ucalyptu* *s* genome. Furthermore, we predicted simple sequence repeats (SSRs) in 45 *Eucalyptus* species genomes (Figure 7b, Table S9). In the SSR submenu, users can query statistical results, positional information, SSR annotation, and SSR sequences, providing researchers with comprehensive tools for browsing and analyzing SSRs, which will support the development of polymorphic SSR markers and molecular‐assisted breeding of eucalyptus. Comprehensive variation data and SSR predictions offer valuable resources for genetic diversity studies, evolutionary analysis, and marker‐assisted selection in *Eucalyptus* species. These findings also lay a foundation for future functional genomics research and the improvement of economically important traits in eucalyptus.

### Functions and tools implemented in EucaMOD

To facilitate data mining and analysis, we incorporated 13 commonly used tools in the Tools module: JBrowse, BLAST, gRNA, Multiple sequence alignment (Muscle), KEGG/GO enrichment, Heatmap, Primer, Gene prediction (GeneWise), Genome alignment (Lastz), Target gene prediction for lncRNAs and miRNAs, TF identification, Transposable element identification, and Motif binding site prediction (Figure 1).

EucaMOD features a custom JBrowse genome browser that enables users to compare and analyze multi‐omics data with graphical views of sequences, chromosome positions, annotations, histone modifications, TF binding sites, SNPs, InDels, SSRs, and more (Figure 1). This integrated visualization tool enhances the ability of researchers to explore complex genomic features and their interactions, facilitating a deeper understanding of the genomics and epigenetic mechanisms underlying eucalyptus biology.

The gRNA tool is an innovative feature designed to select guide RNA (gRNA) sequences for gene editing across 45 eucalyptus genomes. By inputting the gene sequence, researchers can design gRNA sequences that accurately target specific positions within the target gene and obtain information about potential off‐target effects (Figure S5). The tool module includes multiple alignment tools. The BLAST tool, built on NCBI BLAST+ (Ye et al., 2006), is specifically designed for sequence alignment against the gene and protein sequences of all species in EucaMOD. The Muscle (Edgar, 2004) tool enables multiple protein sequence alignment, facilitating comparative analysis of protein families. Additionally, LASTZ (Harris, 2007), a DNA sequence alignment tool optimized for large and complex genomes, is integrated to support high‐accuracy comparisons of genomic sequences among species.

The KEGG/GO enrichment tool facilitates KEGG (Kanehisa, 2000) pathway and GO (Harris et al., 2004) enrichment analysis for target gene sets, providing downloadable results and visual representations of enriched pathways. This tool enables researchers to identify significantly enriched biological processes, molecular functions, cellular components, and metabolic pathways and offers valuable insights into the functional annotation and biological interpretation of gene sets. The Primer tool, which was developed based on Primer3 (Untergasser et al., 2012), is designed to assist users in designing primers for downstream experimental applications by providing customizable parameters, such as melting temperature (Tm), GC content, and amplicon size, ensuring high specificity and efficiency for PCR‐based experiments. GeneWise (Birney et al., 2004), a gene structure prediction tool, uses homologous protein sequences to identify functional similarities in target DNA, enabling accurate gene structure prediction and functional domain annotation. The predicted target genes of miRNAs and lncRNAs can be identified using the Target gene prediction for lncRNAs and miRNAs submenu (Figure S6). Additionally, the Transcription factor identification submenu provides access to a comprehensive collection of 93 TF families among 45 genomes (Table S10).

The Transposable element identification tool in EucaMOD is designed to detect and annotate transposable elements (TEs) among eucalyptus genomes. Utilizing a homology‐based search, this tool identifies different TE classes, including retrotransposons, DNA transposons, and other genetic elements. It provides detailed annotations, such as TE classification, genomic location, and score. The Motif binding site prediction tool in EucaMOD identifies and analyzes TF binding sites within genomic sequences and provides detailed information on motif locations, scores, and matched sequences, facilitating the exploration of transcriptional co‐expression networks. All tools were designed to offer researchers comprehensive capabilities for omics data exploration, functional annotation, and comparative analysis, advancing genomics, and molecular breeding studies in eucalyptus (Figure 1, Table S11).

### Comparative analysis of existing eucalyptus genomic databases

To date, several user‐friendly eucalyptus genomic databases have been established (Table 1). For instance, the EucaMaps database (https://arachne.pierroton.inrae.fr/eucamaps/index.html) integrates 267 QTLs for five traits in six *Eucalyptus* species (*E*. *camaldulensis*, *E. globulus*, *E. nitens*, *E. urophylla*, *E. grandis*, and *E. tereticornis*) and one *Corymbia* species (*C. torelliana* × *C. citriodora*), distributed across 11 linkage groups, into an online visualization and QTL exploration platform (Gion et al., 2014). The EucGenIE database (https://eucgenie.org/) provides genome and transcriptome resources for *E. grandis*, with integrated tools for sequence search, expression analysis, and enrichment analysis (Myburg et al., 2014). The EUCANEXT database (http://bioinfo03.ibi.unicamp.br/eucalyptusdb/) hosts genome sequences for *E. grandis* and *E. camaldulensis*, transcriptome datasets for five species (*E*. *camaldulensis*, *E. globulus*, *E. grandis*, *E. grandis* × *E. urophylla*, and *E. urophylla*), and EST data for six species (*E*. *camaldulensis*, *E. globulus*, *E. grandis*, *E. gunnii*, *E. pellita*, and *E. urophylla*) (Nascimento et al., 2017). It also provides an array of online analytical tools, including GO enrichment, Gbrowse, identification of top expressed genes, heatmap visualization, and expression analysis, enabling basic functional genomics exploration.

**Table 1:** Comparison of eucalyptus genomic databases

Database and its URL	Function	Tools	No. of species
EucaMaps https://arachne.pierroton.inrae.fr/eucamaps/index.html	Genetic map visualization	Eucamaps linkage groups	6
EucGenIE https://eucgenie.org	Genome and transcriptome analysis	Seq Search, Expression Tools, Venn, Enrichment	1
EUCANEXT http://bioinfo03.ibi.unicamp.br/eucalyptusdb	Genome and transcriptome analysis	Go Enrichment, Gbrowse, Top Expressed, Heatmap analysis, Expression analysis	7
EucaMOD http://eucalyptusggd.net/eucamod	Integrative multi‐omics analysis	Global Search, JBrowse, BLAST, gRNA, Muscle, KEGG/Go enrichment, Heatmap, Primer, GeneWise, Lastz, Target gene prediction for IncRNAs and miRNAs, Transcription factor identification, Transposable element identification, Motif binding site prediction	39

Although these early databases have provided valuable genomic and transcriptomic resources, they are limited in species coverage, data types, and recent high‐quality assembly integration. For example, EucGenIE includes only one species, and EUCANEXT does not provide epigenomics or variomics data. In contrast, EucaMOD integrates the most comprehensive eucalyptus multi‐omics dataset to date, covering 39 species. It goes beyond genome and transcriptome data by incorporating epigenomics, variomics, and pan‐proteomics analyses, offering a unique ‘all‐in‐one’ platform. In addition to the tools provided by each omics module, EucaMOD provides an extensive suite of 13 integrated analysis tools, enabling researchers to conduct in‐depth functional genomics studies without leaving the platform. By integrating diverse data types, expanding species coverage, and incorporating state‐of‐the‐art visualization and analytical modules, EucaMOD complements and substantially extends the scope of existing eucalyptus databases, making it a powerful and up‐to‐date resource for molecular breeding and functional genomics research in this economically and ecologically important genus.

### Case study using EucaMOD

APETALA2/ethylene‐responsive factor (AP2/ERF) TFs constitute one of the largest TF families in plants and are closely involved in plant growth, development, and stress responses (Li, Zou, et al., 2025; Wang, Guo, & Yin, 2024). Here, we used the characterization of the ERF gene family in *E. grandis* as a case study to illustrate data retrieval and analysis using the EucaMOD platform. A total of 172 ERF genes were identified and downloaded from *E. grandis* ANBG69807.140 using the Tool–Transcription factor identification module. Subsequently, the Genomics–Gene search module was used to obtain their chromosomal locations, functional domains, and KEGG/GO annotations (Table S12). Function enrichment analyses were conducted using the Tool–KEGG/GO enrichment module, revealing that these genes were predominantly enriched in the KEGG pathways Plant–pathogen interaction, Plant hormone signal transduction, and MAPK signaling pathway–plant and significantly enriched in the GO terms response to temperature stimulus, glycosyl compound metabolic process, and positive regulation of nitrogen compound metabolic process (Figure S7a,b). Subsequently, the Transcriptomics–Gene expression profile module was used to examine ERF gene expression patterns across samples from different research projects, and expression data from temperature‐treated samples were retrieved and downloaded (Table S12), providing a valuable resource for functional studies.

Using the Tool–Heatmap module, we generated expression heatmaps for ERF family genes located on chromosome 6, which had the highest number of ERF genes, in *E. grandis* samples under different temperature treatments. As shown in Figure S7c, several ERF genes exhibited pronounced transcriptional changes in response to low and high temperature stress, displaying distinct upregulation and downregulation patterns. Further analysis revealed that the temperature‐responsive gene LOC104447873 displayed a tissue‐specific expression profile in the Transcriptomics–Gene expression profile module, visualized via eFPs (Figure S7d). LOC104447873 was highly expressed in leaf, root, and xylem tissues but showed lower expression in cotyledon and cambium tissues, suggesting that its stress responsiveness is closely linked to tissue‐specific functions. Using the Transcriptomics–Co‐expression Network module, we constructed the co‐expression network for LOC104447873 and obtained the associated weight values (Figure S8a, Table S13). Detailed gene information and expression data for co‐expressed partners were retrieved using ID‐linked redirection (Table S14), revealing many genes involved in hormone signaling, secondary metabolism, and stress defense pathways. These findings suggest that LOC104447873 has expression patterns associated with temperature adaptation coordinated with functionally related genes and is potentially connected to variation in tissue‐specific transcriptional programs in response to environmental signals.

We systematically explored LOC104447873 across multiple omics modules in EucaMOD. In the Pan‐proteomics module, LOC104447873 was classified as a core gene family member, indicating its conserved presence across all analyzed species. In the Variomics module, whole‐genome resequencing of *E. grandis* revealed genetic variations in LOC104447873, including a SNP with a G‐to‐A substitution and an InDel in which ‘AGAGGAG’ was deleted to ‘A’ (Table S15, Figure S8b–d). Using the Epigenetics–TF binding module, we detected a MYB2 binding site (Chr6: 2201976–2202082) within LOC104447873, suggesting that MYB2 might influence its transcription (Table S16, Figure S8e). The Comparative genomics–Homologs module revealed that LOC104447873 contains both orthologous and paralogous genes across different *E. grandis* genotypes, suggesting that it may play conserved roles among genotypes. This module also provides a phylogenetic tree depicting homologous genes of LOC104447873 in different *E. grandis* genotypes (Table S17, Figure S8f). Finally, the global search function on the EucaMOD homepage enables users to retrieve and download all omics information for LOC104447873, as exemplified in Figure S1. In addition to the previously discussed modules, the Tools module offers a global search on the homepage and 13 interactive online tools that support comprehensive, multidimensional omics analyses (Figure 1), facilitating convenient execution of subsequent analyses for users.

## CONCLUSION

Compared to other eucalyptus genomics databases, EucaMOD offers several unique advantages, including: (1) Comprehensive multi‐omics coverage: Currently, EucaMOD is the most comprehensive eucalyptus multi‐omics database, encompassing all publicly available *Eucalyptus* species. To effectively utilize multi‐omics datasets to support eucalyptus research, we integrated and visualized a vast array of genomics, comparative genomics, transcriptomics, epigenomics, and variomics datasets. (2) The first eucalyptus‐focused pan‐proteomics and epigenomic database: EucaMOD offers valuable resources for eucalyptus genomics and molecular breeding research, promoting functional genomics exploration. (3) Efficient data mining platform: EucaMOD provides researchers with a user‐friendly and efficient analytical platform that features a variety of online multi‐omics data analysis tools. All tools are accompanied by detailed user manuals. (4) Innovative tool—gRNA: EucaMOD offers a unique gRNA design tool that enables the precise targeting of specific locations within genes and provides information on potential off‐target effects. (5) Cross‐species gene homology analysis: EucaMOD facilitates the exploration of gene homology relationships among species, allowing for cross‐species comparisons of gene functions and multi‐omics features and aiding the in‐depth investigation of evolutionary relationships among species. (6) User‐friendly data integration: EucaMOD's Search tool and JBrowse interface support the seamless integration and cross‐referencing of multi‐omics datasets in an interactive and user‐friendly manner.

In summary, EucaMOD is the most comprehensive multi‐omics database for eucalyptus, encompassing the latest genome, comparative genome, pan‐proteome, transcriptome, epigenome, and variation data and providing practical analysis tools. It is a valuable online resource for eucalyptus functional genomics and molecular breeding research. In the future, with the release of more multi‐omics datasets, such as genomics, metabolomics, and proteomics datasets, in *Eucalyptus* species, we will continue to update EucaMOD. Moreover, a future goal of EucaMOD is to leverage machine learning to combine multi‐omics data with artificial intelligence‐driven breeding, enabling precision breeding for eucalyptus. We welcome opinions and suggestions from eucalyptus researchers all over the world to enhance and improve EucaMOD.

## MATERIALS AND METHODS

### Data sources

To establish a comprehensive multi‐omics dataset for eucalyptus, the following publicly available data were gathered, analyzed, and integrated: genomics (45 genomes from 39 species), transcriptomics (870 RNA‐seq and 17 miRNA‐seq datasets), epigenomics (52 datasets, including chromatin immunoprecipitation sequencing [ChIP‐seq], DNA affinity purification sequencing [DAP‐seq], and DNase I hypersensitive sites sequencing [DNase‐seq]), and 1219 variation datasets were collected from NCBI (https://www.ncbi.nlm.nih.gov/), Phytozome (https://phytozome‐next.jgi.doe.gov/), Figshare (https://figshare.com/), NGDC (https://ngdc.cncb.ac.cn/), Ensembl Plants (https://plants.ensembl.org/), and TAIR (https://www.arabidopsis.org/) databases. The detailed volumes and sources are shown by EucaMOD.

### Database construction

EucaMOD was deployed and operated on Ubuntu 20.04 (https://ubuntu.com/), using MySQL 8.0.40 (https://www.mysql.com/) for data storage and management, Slick 3.3.2 (Parashar et al., 2006) as the middleware layer to optimize query performance and enable efficient data retrieval, AKKA 2.6.4 (Srirama et al., 2021) (https://doc.akka.io) as the web server framework for handling concurrent and distributed tasks, and Scala 2.13.2 (https://www.scala‐lang.org/) with SBT 1.3.9 (https://www.scala‐sbt.org/) for system development and compilation, ensuring high performance and scalability. Bootstrap 5.1.3 (https://getbootstrap.com), Highcharts 9.3.2 (https://www.highcharts.com/), jQuery 3.6.0 (https://jquery.com/), and PlayFramework 2.8.7 (https://www.playframework.com/) were used to design and implement the website interfaces. In addition, JBrowse 2.0 (Buels et al., 2016) (https://www.jbrowse.org), a powerful genome browser tool, was used to visualize multiple sets of scientific data, providing users with a friendly interactive page. EucaMOD was rigorously tested on common web browsers, including Firefox, Google Chrome, and Internet Explorer, and showed stable performance.

### Genome annotation

Following the published genome annotation pipeline (Haas et al., 2008; Hoff et al., 2019; Holt & Yandell, 2011; Thibaud et al., 2016), structural annotation was performed on 15 genomes, including *E*. *camaldulensis*, *E*. *cloeziana*, *E*. *urophylla*, *E*. *globulus*, and *E*. *grandis*, which are commonly used by our research group, and *A*. *floribunda*, *C*. *calophylla*, *C*. *maculata*, *E*. *melliodora*, *E*. *polybractea*, *E*. *regnans*, and *E*. *sideroxylon*, which had not been annotated by June 2024. Functional annotation and ncRNA prediction were conducted for all 45 genomes. The sequences and annotation information of all genomes were integrated into JBrowse for comprehensive visualization and analysis.

### Comparative genomics and pan‐proteomics analyses

Orthologous and paralogous genes across 39 species (45 genomes) were identified using OrthoFinder 2.5.4 (Emms & Kelly, 2019). Collinear blocks were identified using MCScanX 1.1.11 (Wang et al., 2012) with default parameters. Additionally, a phylogenetic tree was constructed using MEGA‐X (Kumar et al., 2018) based on single‐copy orthologous gene families among *Eucalyptus* species.

### RNA‐seq and miRNA‐seq analysis

After quality control using Fastp 0.20.0 (Chen et al., 2018), the reads of each *Eucalyptus* species were aligned to their respective reference genomes using HISAT2 2.0.5 (Table S5 and S6) (Kim et al., 2019), and gene expression was quantified using StringTie 1.3.6 (Pertea et al., 2015). Transcripts longer than 200 nt with more than two exons were selected as lncRNA candidates containing CNCI 2.0, CPAT 3.0.0, CPC 2 0.1, and Pfam 35.0. In miRNA‐seq analysis, sRNAminer 1.1.2 (Li et al., 2024) was employed for miRNA identification, target prediction, and expression analysis. A co‐expression network was constructed using WGCNA 1.70.3 (Langfelder & Horvath, 2008), which measured the correlation of gene expression patterns across samples using a weighted correlation matrix and generated modules based on their co‐expression patterns. The resulting co‐expression network was visualized using Cytoscape 3.6.1 (Su et al., 2014).

### Epigenomic data processing

For ChIP‐seq, DAP‐seq, and DNase‐seq, Trimmomatic 0.36 (Bolger et al., 2014) was used to remove adapter sequences and filter out low‐quality reads. The clean data obtained from germplasm resources were aligned to *E. grandis* reference genome assembly (GCA_016545825.1) using Bowtie2 2.3.2 (Langdon, 2015) with default parameters (Table S7). Picard 2.19 was used to remove PCR‐duplicated reads, and peaks were called using the callpeak module of MACS2 2.1.2 (Feng et al., 2012).

### Genomic variation detection

After using Trimmomatic 0.36 (Bolger et al., 2014) to control the raw data quality, BWA 0.7.12 (Jo & Koh, 2015) was used to map the clean data to the respective reference genome of each *Eucalyptus* species (Table S8). Then, Samtools 1.13 (Li et al., 2009) was used to transform and sort the mapping results, and GATK 4.1.5.0 (Summa et al., 2017) was used to implement the HaplotypeCaller‐based unified genotyping approach for SNP and InDel calling.

### Tool development

EucaMOD integrates a customized genome browser based on JBrowse 2.0 (Buels et al., 2016), enabling users to compare and analyze various omics datasets. The BLAST 2.12.0+ (Ye et al., 2006) tool was implemented for sequence alignment, supporting three alignment modes: blastn gene, blastn genome, and blastp protein. The gRNA tool provided a comprehensive analysis of guide sequences using CRISPR‐P 2.0 (Liu et al., 2017) technology. MUSCLE 5.1 (Edgar, 2004), one of the most efficient multiple sequence alignment programs, consistently outperforms CLUSTALW (Thompson et al., 1994) in both accuracy and speed and is capable of aligning hundreds of sequences within seconds. KEGG (Kanehisa, 2000) and GO (Harris et al., 2004) enrichment analysis tools were developed to integrate genomic functional annotation data, offering gene function classification and pathway enrichment analysis to enable users to gain deeper insights into the biological functions of genes and their roles in metabolic pathways. The Heatmap tool can be used to customize expression data visualization through standardized data processing and algorithm optimization. Primer3 2.6.1 (Untergasser et al., 2012), a widely used program for designing PCR primers, was embedded in EucaMOD. GeneWise 2.4.1 (Birney et al., 2004) compares protein sequences with genomic DNA sequences. LASTZ 1.04.00 (Harris, 2007) is a highly efficient tool for genome alignment that was specifically designed to compare two DNA sequences and identify similarities and differences. The TF identification tool leveraged iTAK 2.0.2 (Zheng et al., 2016) to provide detailed information on TFs across eucalyptus genomes. EDTA 2.0.0 (Su et al., 2021) is a toolkit for de novo annotation of TEs across entire genomes and for evaluating the quality of existing TE library annotations. Find individual motif occurrences (FIMO), a module within MEME Suite 5.5.0 (Bailey et al., 2009), is used to scan and identify functional motif occurrences in DNA or protein sequences. All tools mentioned above can be referenced in Table S11.

The Supporting Information provides comprehensive methodological details encompassing genome annotation analysis, comparative genomics and pan‐proteome analyses, RNA‐seq and miRNA‐seq analyses, epigenomic data processing, and genomic variation detection.

## AUTHOR CONTRIBUTIONS

ZFX and ML conceived and designed the project. ML and YC, collected and analyzed, built the database. ML, YC, WW, YM, JW, XG, JX, YF, GS, HH, KL, JN and ZFX wrote and revised the manuscript. All authors read and approved the final manuscript.

## CONFLICT OF INTEREST

The authors declare no competing interests.

## Supporting information


**Figure S1.** Multi‐omics data visualization outputs from the “Search” tool for gene LOC104415177 from *Eucalyptus grandis*, a homolog of *Arabidopsis thaliana* NAC domain containing protein 90 (ANAC090).
**Figure S2.** Overview of the Species module in EucaMOD.
**Figure S3.** Phylogenetic tree of *Eucalyptus* species based on single‐copy orthologous groups using MEGA.
**Figure S4.** ncRNA expression and visualization in EucaMOD.
**Figure S5.** Precise CRISPR target selection for gene editing in the “gRNA” tool.
**Figure S6.** Network analysis of miRNA and its multiple target genes in the “Target prediction” submenu.
**Figure S7.** Functional enrichment and expression profiles of ERF gene family members in *Eucalyptus grandis* ANBG69807.140.
**Figure S8.** Genetic variation, TF binding‐site features, and evolutionary relationships of LOC104447873 in *Eucalyptus grandis* ANBG69807.140.

**Table S1.** Data source of genome assemblies in EucaMOD.
**Table S2.** Statistics of the gene function annotation of genomes in EucaMOD.
**Table S3.** Statistics of 15 genome re‐annotations in EucaMOD.
**Table S4.** Gene family statistics for the pan‐proteome in EucaMOD.
**Table S5.** Summary of RNA‐seq datasets in EucaMOD.
**Table S6.** Summary of miRNA‐seq datasets in EucaMOD.
**Table S7.** Summary of epigenetics datasets in EucaMOD.
**Table S8.** Summary of variomics datasets in EucaMOD.
**Table S9.** SSR statistics for all genomes in EucaMOD.
**Table S10.** Summary of transcription factors among 45 genomes in EucaMOD.
**Table S11.** Databases and tools referenced in EucaMOD.
**Table S12.** Expression and gene information of ERF family members in *Eucalyptus grandis* ANBG69807.140.
**Table S13.** Co‐expression of LOC104447873 in *Eucalyptus grandis* ANBG69807.140.
**Table S14.** Expression levels and gene information of genes co‐expressed with LOC104447873 in *Eucalyptus grandis* ANBG69807.140.
**Table S15.** SNP and InDel of LOC104447873 detected by whole‐genome sequencing of *Eucalyptus grandis*.
**Table S16.** DAP‐seq of *Eucalyptus grandis* identified MYB2 binding peaks within the LOC104447873 genomic region.
**Table S17.** Identification of LOC104447873 homologous genes among *Eucalyptus grandis* genotypes.

## Data Availability

All of the genome, datasets, tools, and tutorials can be accessed from EucaMOD (http://eucalyptusggd.net/eucamod). All users have free access without logging in.
