# Supplementary material for: EucaMOD: a comprehensive multi‐omics database for functional genomics research and molecular breeding of fast‐growing eucalyptus trees
Source: Plant J. 2025 Dec 15;124(5):e70603. doi: 10.1111/tpj.70603 (PMC12704907; doi:10.1111/tpj.70603)
Supplement: Supplementary file 1 — Figure S1. Multi‐omics data visualization outputs from the “Search” tool for gene LOC104415177 from Eucalyptus grandis, a homolog of Arabidopsis thaliana NAC domain containing protein 90 (ANAC090). Figure S2. Overview of the Species module in EucaMOD. Figure S3. Phylogenetic tree of Eucalyptus species based on single‐copy orthologous groups using MEGA. Figure S4. ncRNA expression and visualization in EucaMOD. Figure S5. Precise CRISPR target selection for gene editing in the “gRNA” tool. Figure S6. Network analysis of miRNA and its multiple target genes in the “Target prediction” submenu. Figure S7. Functional enrichment and expression profiles of ERF gene family members in Eucalyptus grandis ANBG69807.140. Figure S8. Genetic variation, TF binding‐site features, and evolutionary relationships of LOC104447873 in Eucalyptus grandis ANBG69807.140. [file TPJ-124-0-s001.docx]

**Detailed methods**

**Genome annotation analysis**

The basic information on genome assembly and genes was obtained from the genome and annotation files. Following the published genome annotation pipeline (Haas *et al.*, 2008, Holt and Yandell, 2011, Thibaud *et al.*, 2016, Hoff *et al.*, 2019), structural annotation was performed on 15 genomes by June 2024. A species-specific repeat library was built using RepeatModeler 2.03 (https://www.repeatmasker.org/) and combined with the RepBase 21.12 database (Bao *et al.*, 2015) to comprehensively identify repetitive elements across the genome using RepeatMasker 4.13 (Flynn *et al.*, 2020) with default parameters. Four tools were used for *de novo* gene prediction: GeneMark-ET 4.38 (Borodovsky and McIninch, 1993) with default parameters; SNAP 7.0 (Korf, 2004) with default parameters; AUGUSTUS 3.4.0 (Stanke *et al.*, 2006) --species --gff3=on; and BRAKER 2.1.6 (Bruna *et al.*, 2021) run via braker.pl --genome genome.fa --bam rnaseq.bam --species=eucalyptus. Homology-based predictions were performed with GenomeThreader 1.7.3 (Myburg *et al.*, 2014) with default parameters by aligning protein sequences from *Arabidopsis thaliana*, *Oryza sativa*, *Populus trichocarpa*, and *Eucalyptus* (Myburg *et al.*, 2014). For transcriptome-based evidence, StringTie 2.2.1 (Pertea *et al.*, 2015) was utilized for the *de novo* assembly of RNA-Seq data from *Eucalyptus*. Trinity 2.8.5 (Haas *et al.*, 2003) with --genome_guided_bam rnaseq.bam was employed for genome-guided transcriptome assembly. TransDecoder 5.5.0 (Haas *et al.*, 2013) was then applied to predict the coding sequences from the assembled transcripts. RNA-Seq data were integrated using PASA 2.5.2 (Haas *et al.*, 2008) via Launch_PASA_pipeline.pl -c alignAssembly.config -R -g genome.fa -t transcripts.fa --ALIGNERS blat,gmap to construct a comprehensive transcriptome database. All evidence types—*de novo* predictions, homology-based annotations, and transcripts—were integrated in MAKER3 3.1.3 (Cantarel *et al.*, 2008) using maker -base eucalyptus_annotation -genome genome.fa -est transcripts.fa -protein proteins.fa -rm_gff repeatmasker_out.gff3 -pred_gff predictions.gff3 to optimize gene model prediction. EVM 2.1.0 (Haas *et al.*, 2008) --weights weights.txt --gene_predictions denovo_preds.gff3 --transcript_alignments transcript_out.gtf --protein_alignments proteins.gff3 was subsequently used to generate weighted consensus gene models. A final round of annotation updates was performed using PASA 2.5.2 (Haas *et al.*, 2008) with -c alignAssembly.config -A -g genome.fasta -t all_transcripts.fasta –annots EVM.gff3 to refine gene structures and predict alternative splicing events and 5’ and 3’ untranslated regions. Gene function prediction was conducted for all 46 genomes. Gene functional annotation was performed by querying multiple databases, including Swiss-Prot (Apweiler *et al.*, 2004), NCBI-NR (Pruitt *et al.*, 2007), Pfam (Mistry *et al.*, 2021), Gene Ontology (GO) (Ashburner *et al.*, 2000), and Kyoto Encyclopedia of Genes and Genomes (KEGG) (Kanehisa *et al.*, 2004).

Non-coding RNA prediction was conducted for all 46 genomes. Candidate lncRNAs were identified based on full-length transcript assemblies generated with the Iso-Seq data. Transcripts with a length ≥200 nucleotides and containing at least 2 exons were initially chosen as lncRNAs. These were further filtered using computational tools with coding potential assessment capabilities, including CNCI 2.0 (Sun *et al.*, 2013), CPAT 3.0.0, CPC2 0.1 (Kang *et al.*, 2017), PLEK 2 (Li *et al.*, 2024a), and Pfam 35.0 (Mistry *et al.*, 2021), with default parameters, to reliably distinguish non-coding transcripts from protein-coding transcripts. tRNAs were predicted using tRNAscan-SE 2.0.12 (Lowe and Eddy, 1997) with default parameters. snRNAs and miRNAs were identified by aligning sequences against the Rfam database (Kalvari *et al.*, 2018) through Infernal 1.1.4 (Nawrocki and Eddy, 2013) with cmscan --cut_ga --rfam --nohmmonly --tblout infernal.tblout Rfam.cm genome.fa. rRNAs were annotated using BLAST 2.14.0 (Kent, 2002) by aligning the eucalyptus genome against known rRNA sequences to accurately locate rRNA regions.

**Comparative genomics and pan-proteome analyses**

Orthologous and paralogous genes were identified in 39 species (45 genomes) using OrthoFinder 2.5.4 (Emms and Kelly, 2019) with parameters -M msa -S diamond -T fasttree. Collinear blocks were identified using MCScanX 1.1.11 (Wang *et al.*, 2012) with -e 1e-5 -s 5 -m 25. A phylogenetic tree was constructed using MEGA-X 10.2.6 (Kumar *et al.*, 2018) applying the maximum likelihood method and 1000 bootstrap replicates based on single-copy orthologous gene families among eucalyptus species.

**RNA-seq and miRNA-seq analyses**

After quality control using fastp 0.20.0 (Chen *et al.*, 2018) with default parameters, the reads were aligned to the reference genome using HISAT2 2.0.5 (Kim *et al.*, 2019) with --dta –new-summary --rna-strandness RF. Subsequently, Samtools 1.13 (Li *et al.*, 2009) was further used to convert SAM format into BAM format. Gene expression was quantified using StringTie 1.3.6 (Pertea *et al.*, 2015) with -e -B -G ref_annotation.gtf. For miRNA-seq analysis, sRNAminer 1.1.2 (Li *et al.*, 2024b) with default parameters was employed in miRNA identification, target prediction, and expression analysis. A co-expression network was constructed using WGCNA 1.70.3 (Langfelder and Horvath, 2008). The TPM expression matrix was log_2_-transformed and quantile-normalized across samples using the preprocessCore package. Genes with zero variance or expressed in fewer than 50% of samples were excluded from the analysis. The network was built with the parameters power = 12, minModuleSize = 30, and mergeCutHeight = 0.25 to measure the correlation of gene expression patterns across samples using a weighted correlation matrix and to group genes into modules based on their co-expression patterns. The resulting co-expression network was visualized using Cytoscape 3.6.1 (Su *et al.*, 2014) with default layout settings.

**Epigenomic data processing**

For ChIP-seq, DAP-seq, and DNase-seq, Trimmomatic 0.36 (Bolger *et al.*, 2014) with default parameters was used to remove adapter sequences and filter out low-quality reads. The clean data obtained from germplasm resources were aligned to the reference genome assemblies using Bowtie2 2.3.2 (Langdon, 2015) with default parameters. Picard 2.19 with REMOVE_DUPLICATES=true was used to remove PCR-duplicated reads, and peaks were called using the callpeak module of MACS2 2.1.2 with -nomodel -q 0.05 --extsize 200 --keep-dup all -B --call-summit (Feng *et al.*, 2012).

**Genomic variation detection**

After using Trimmomatic 0.36 (Bolger *et al.*, 2014) with default parameters to control the raw data quality, BWA 0.7.12 (Jo and Koh, 2015) was used to map the clean data to the reference genome. Samtools 1.13 (Li *et al.*, 2009) was then used to transform and sort the mapping results, and GATK 4.1.5.0 (Summa *et al.*, 2017) with parameters QUAL < 30.0, MQ < 50.0, QD < 2 was used to implement a unified genotype approach for SNP and InDel calling.

**S****upplementary figures**


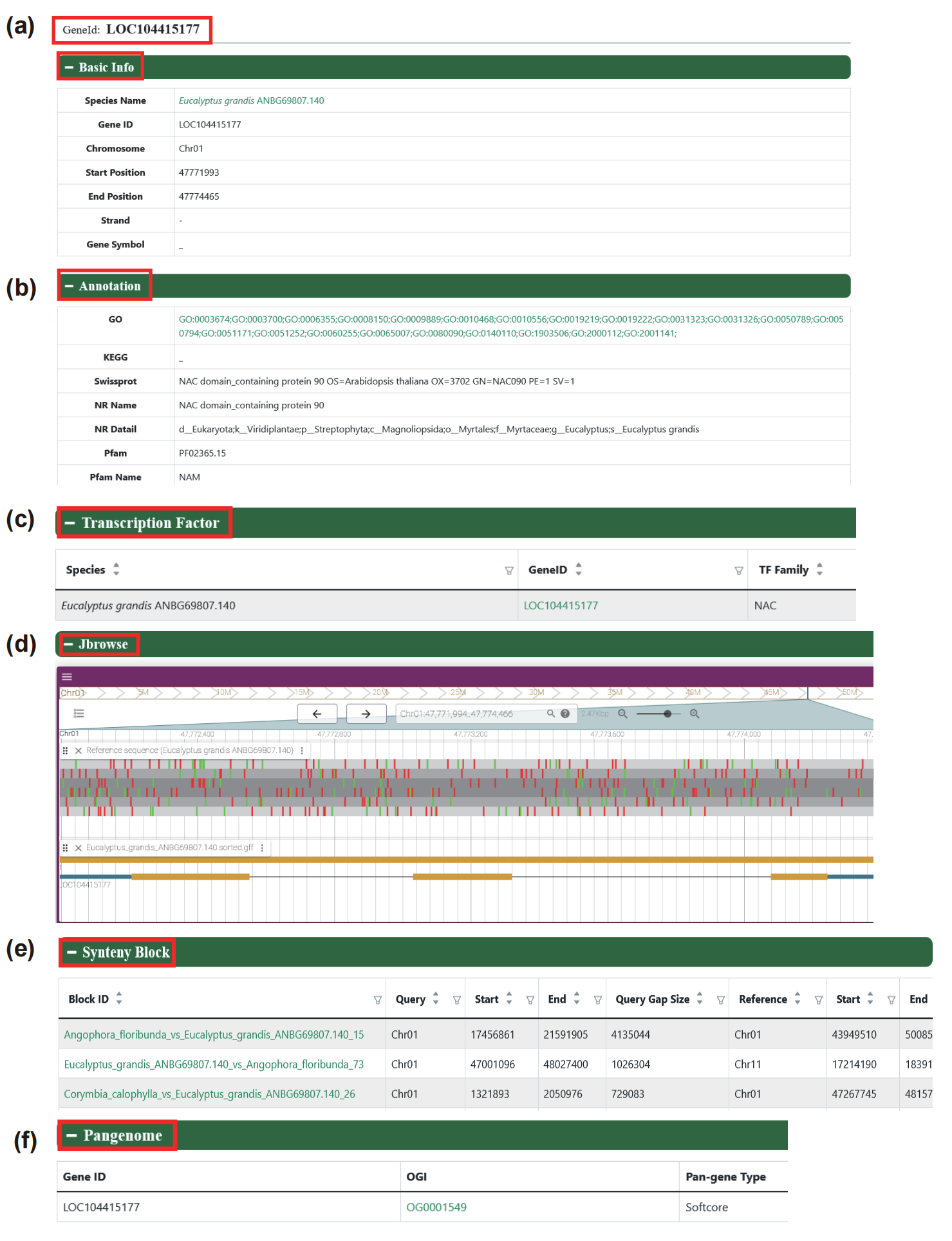


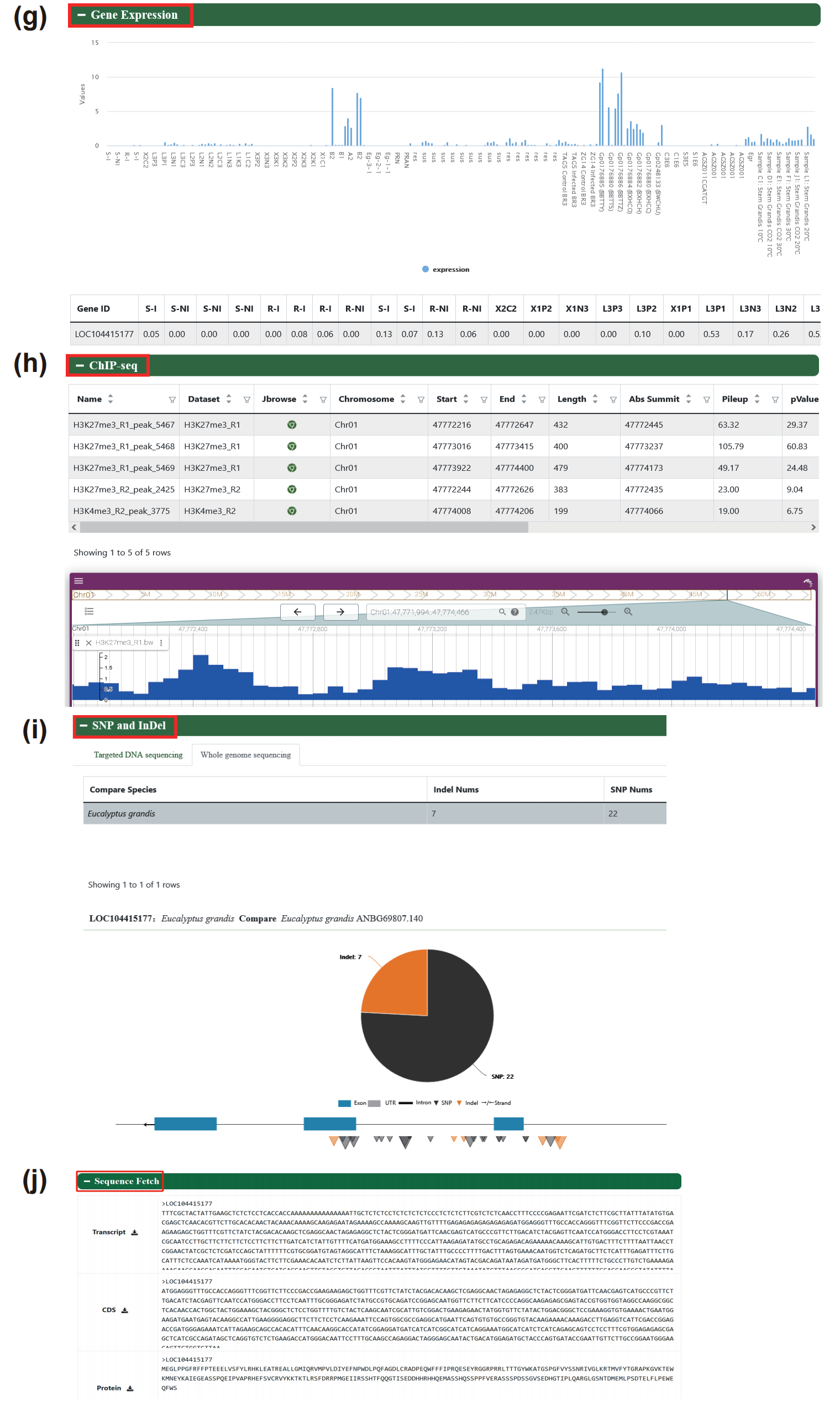


**Figure S1. Multi-omics data visualization outputs from the “Search” tool for gene LOC104415177 from *Eucalyptus grandis*, a homolog of *Arabidopsis thaliana* NAC domain containing protein 90 (ANAC090).**

(a) Genomic feature summary.

(b) Functional annotation overview.

(c) Identification of LOC104415177, a member of the NAC transcription factor family.

(d) Genome browser tracks (JBrowse) displaying the genomic context and annotations.

(e) Syntenic block between *Eucalyptus grandis* and other eucalyptus genomes.

(f) Pan-proteome classification in the gene family repertoire.

(g) Transcript abundance profiles across tissues.

(h) Epigenomic landscape visualization (histone modification patterns).

(i) Genomic variation analysis showing the SNP/InDel distribution.

(j) Sequence retrieval for transcripts, coding sequences (CDSs), and protein products.


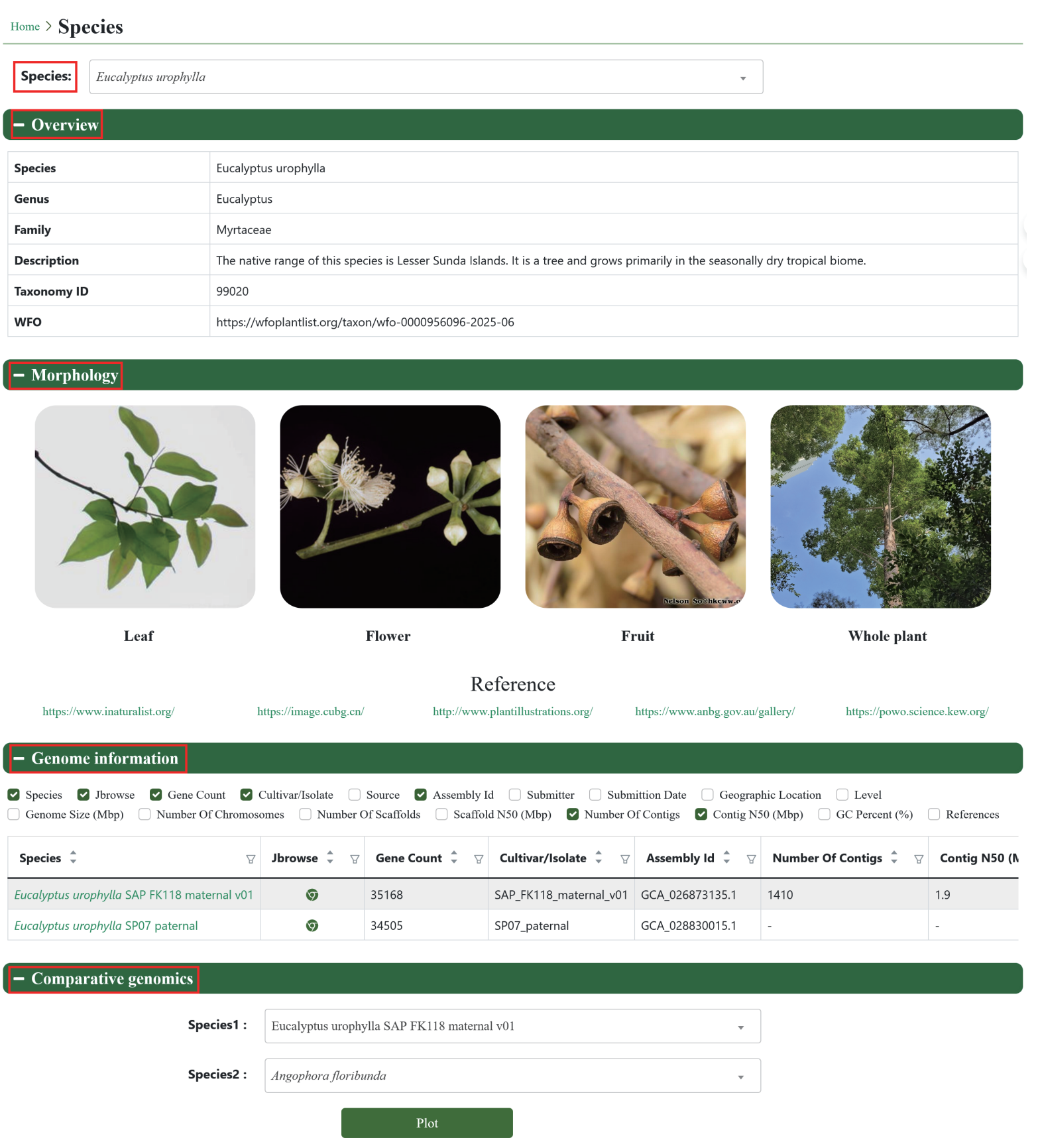
 **Figure S2. Overview of the Species module in EucaMOD.**


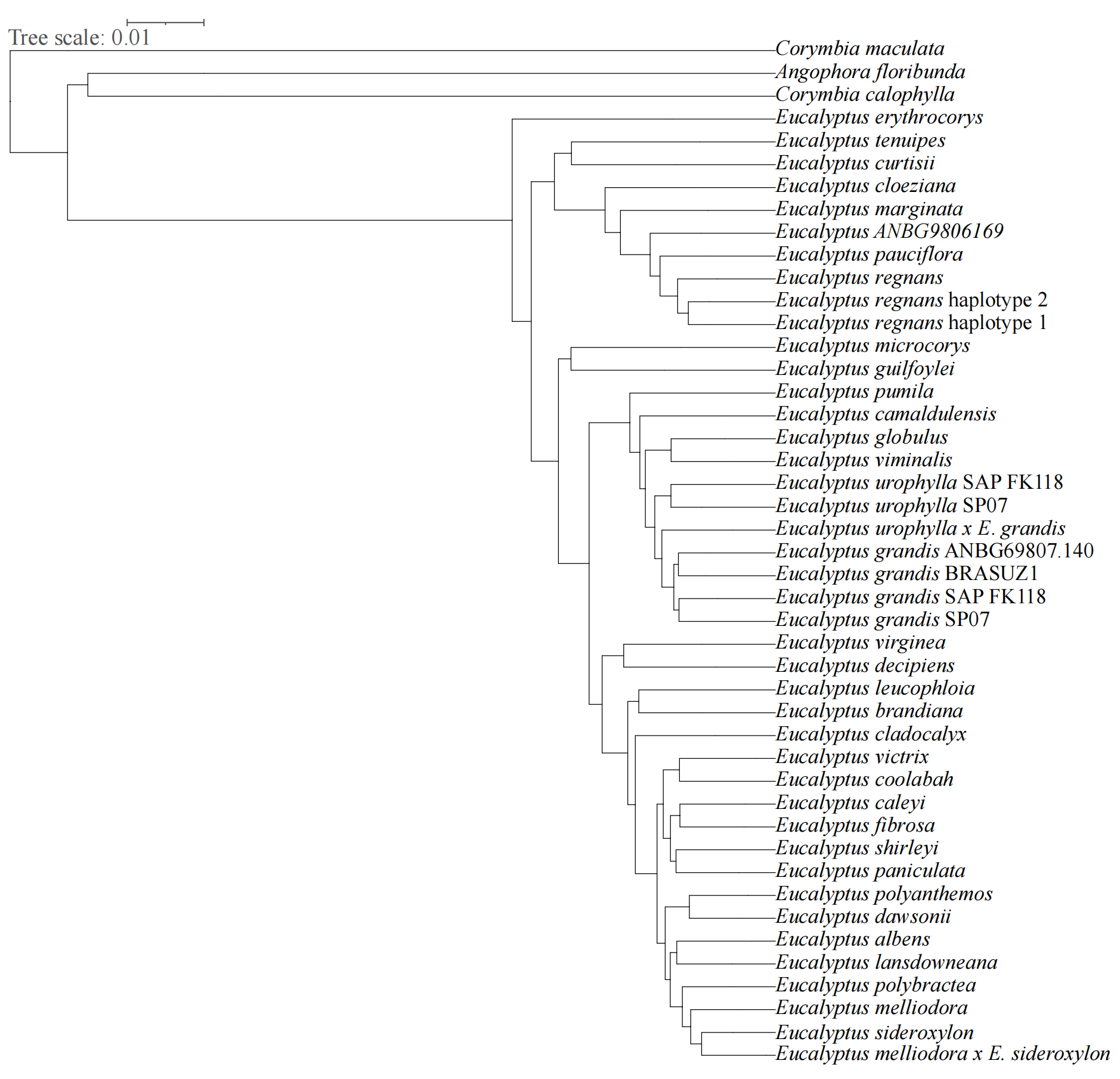


**Figure S3. Phylogenetic tree of eucalyptus species based on single-copy orthologous groups using MEGA.**


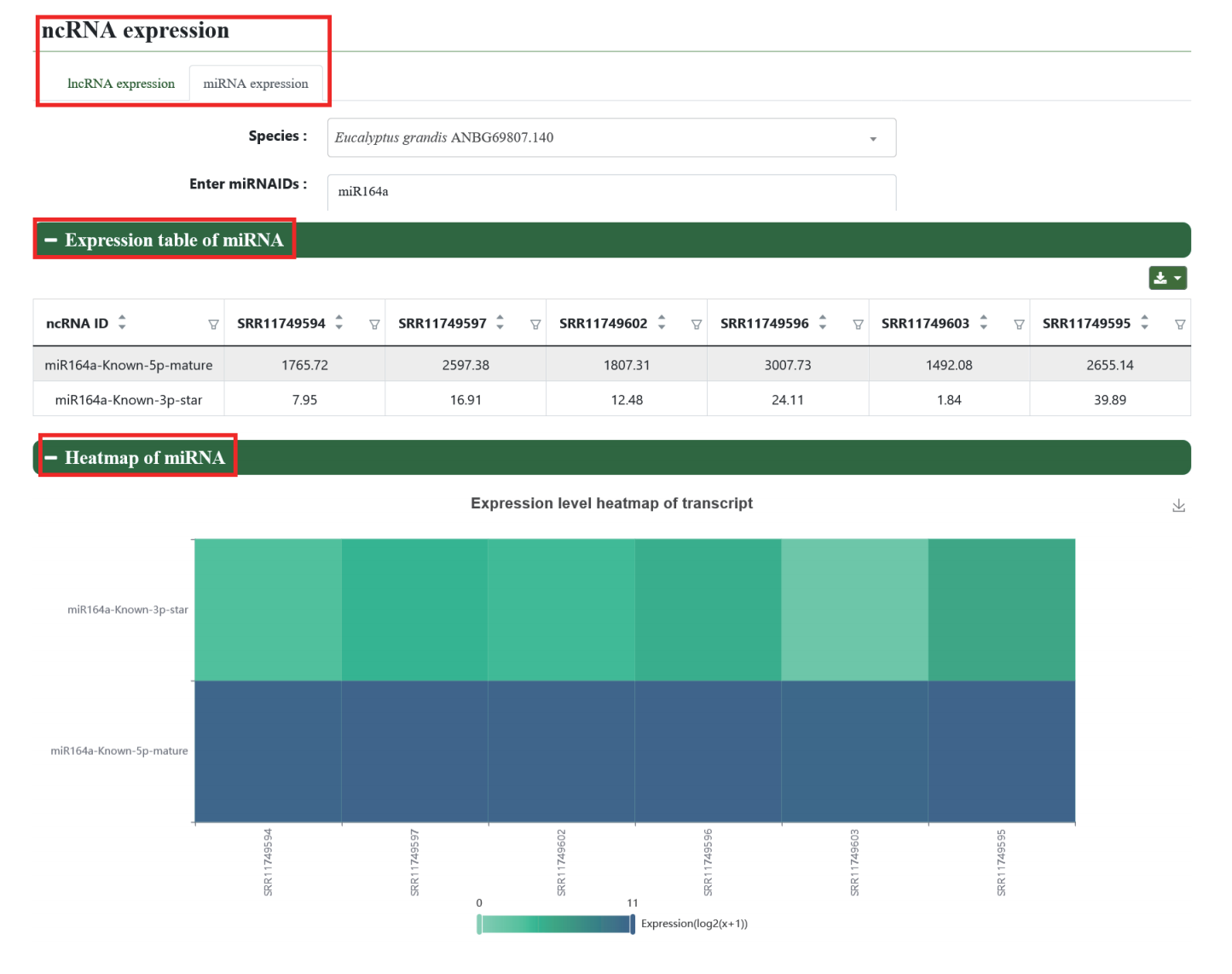


**Figure S4. ncRNA expression and visualization in EucaMOD.**

**
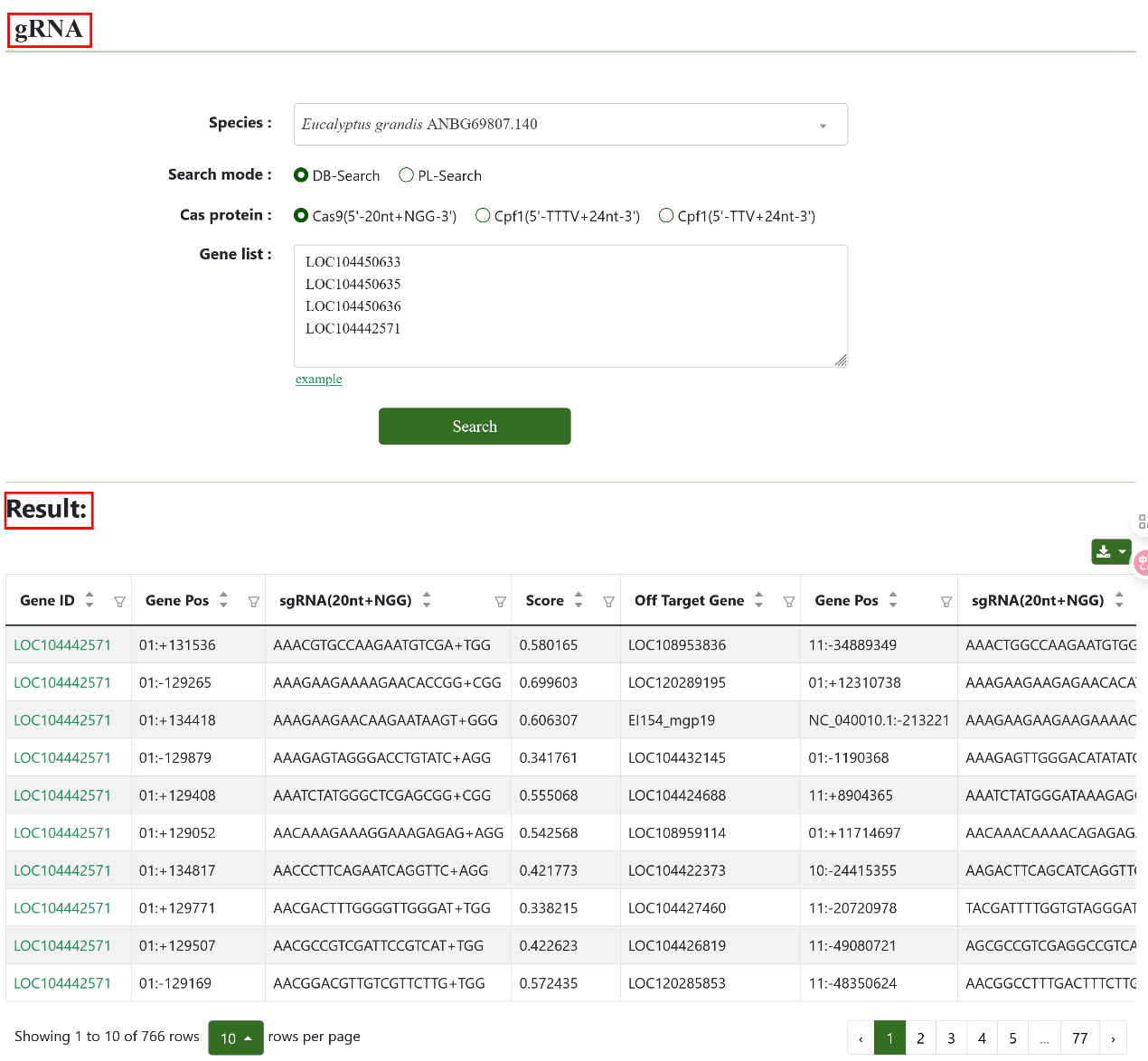
**

**Figure S5. Precise CRISPR target selection for gene editing in the “gRNA” tool.**


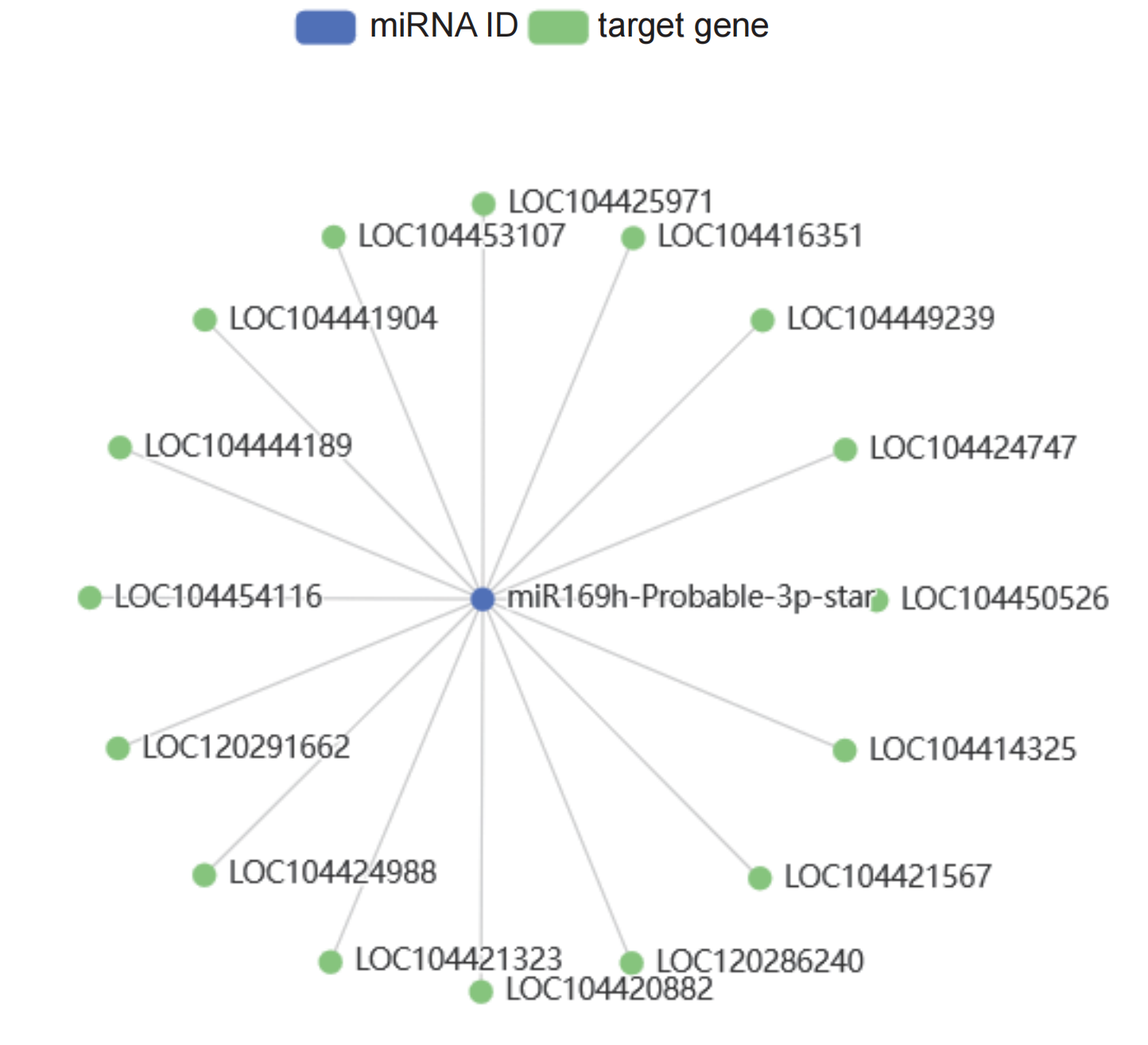


**Figure S6. Network analysis of miRNA and its multiple target genes in the “Target prediction” submenu.**


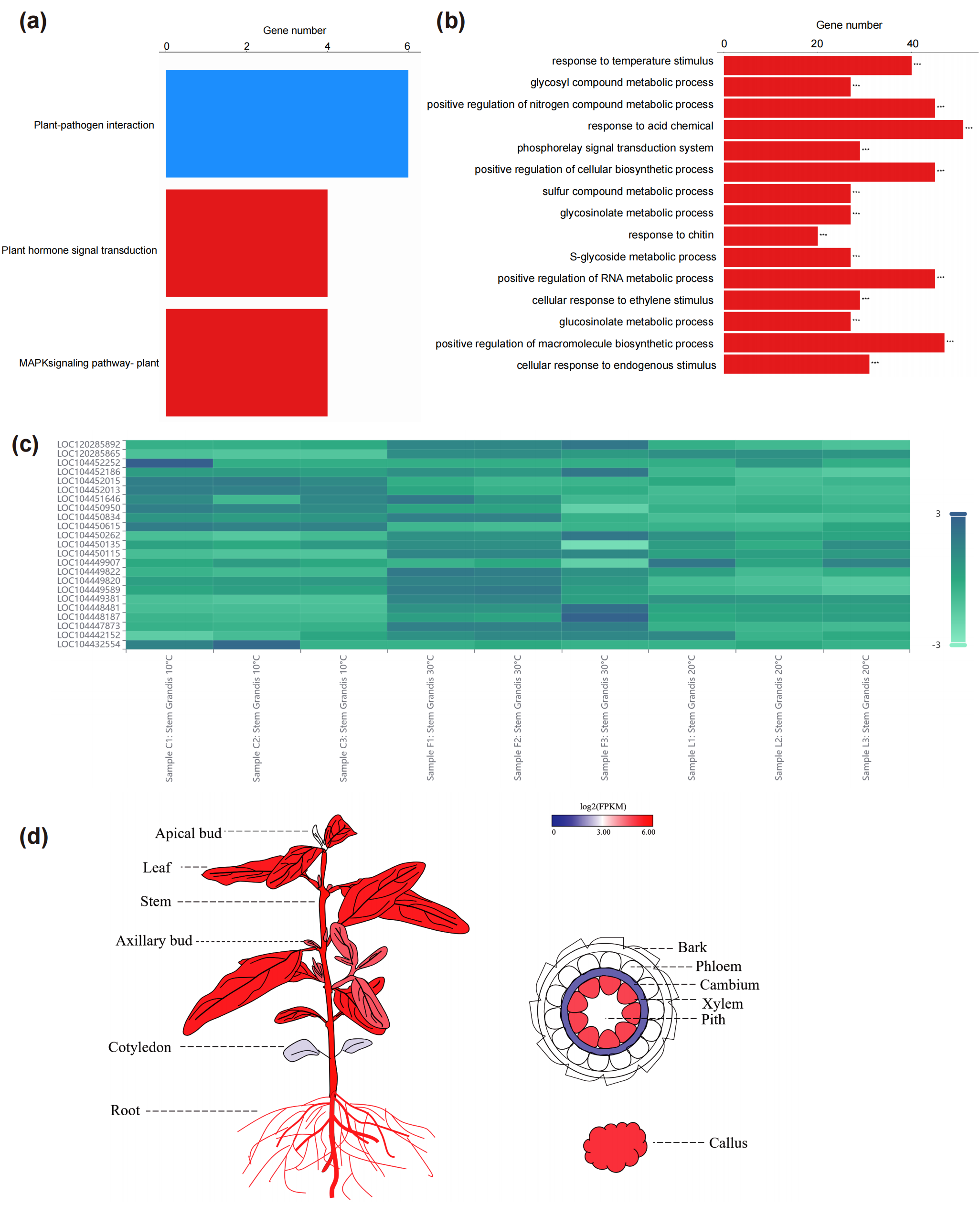


**Figure S7.** **Functional enrichment and expression profiles of ERF gene family members in *Eucalyptus grandis* ANBG69807.140.**

(a) KEGG pathway enrichment analysis of ERF gene family members.

(b) GO enrichment analysis of ERF gene family members.

(c) Heatmap showing the expression levels of ERF genes on chromosome 6 under normal, low, and high temperature conditions.

(d) Expression profile of LOC104447873 among tissues, visualized using the eucalyptus eFP browser.


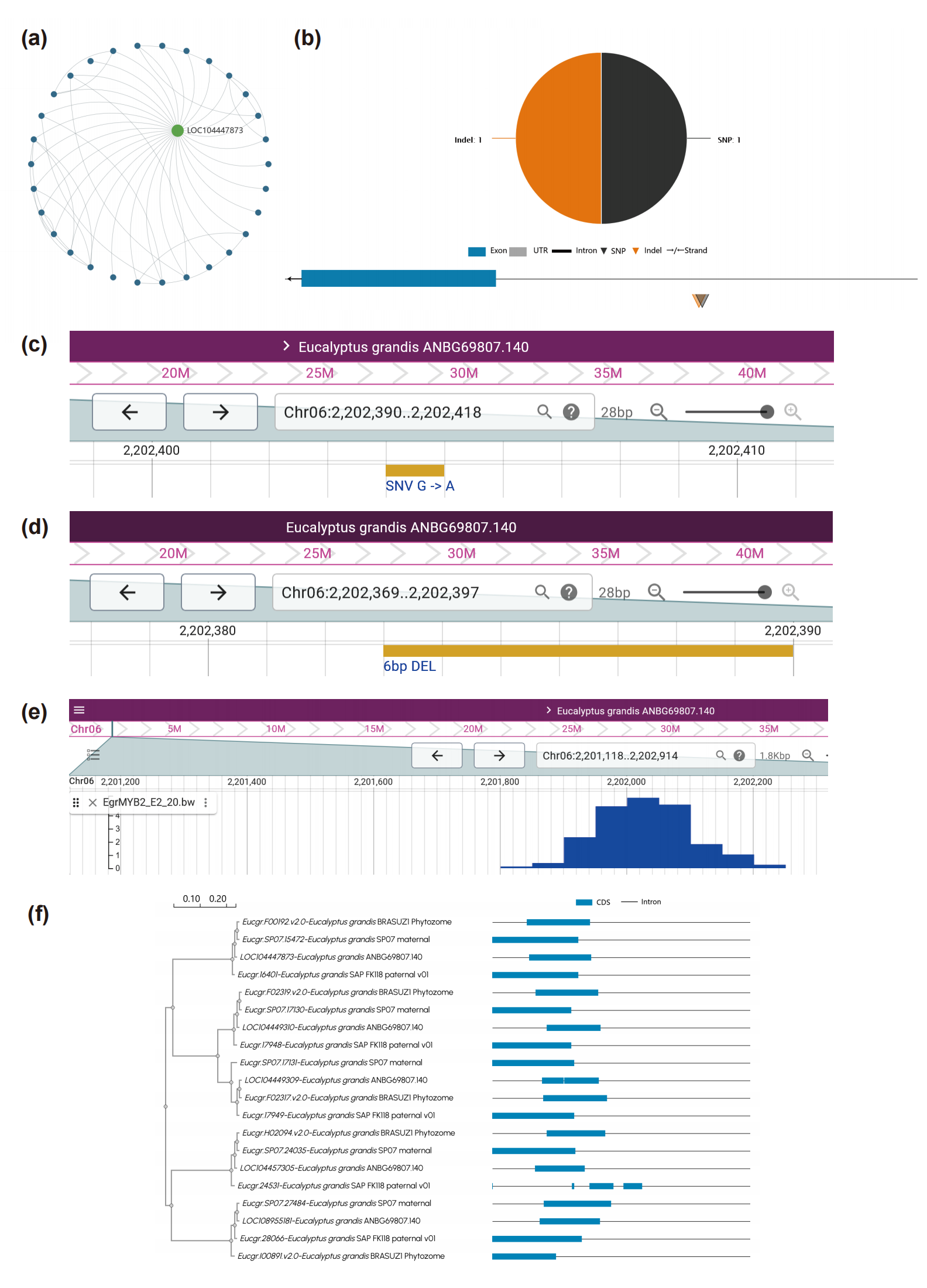


**Figure S8. Genetic variation, TF binding-site features, and evolutionary relationships of LOC104447873 in *Eucalyptus grandis* ANBG69807.140.**

(a) Co-expression network of LOC104447873.

(b) Statistics of genetic variations in LOC104447873 and the genomic positions of SNPs and InDels based on whole genome sequencing data.

(c) Visualization of SNPs within LOC104447873 using JBrowse.

(d) Visualization of InDels within LOC104447873 using JBrowse.

(e) Visualization of MYB2 binding sites within LOC104447873 identified by DAP-seq using JBrowse.

(f) Phylogenetic tree of LOC104447873 homologs across *Eucalyptus grandis* genotypes.

**References**

**Apweiler, R., Bairoch, A., Wu, C.H., Barker, W.C., Boeckmann, B., Ferro, S., Gasteiger, E., Huang, H., Lopez, R., Magrane, M., Martin, M.J., Natale, D.A., O'Donovan, C., Redaschi, N. and Yeh, L.S.** (2004) UniProt: the Universal Protein knowledgebase. *Nucleic Acids Res*, **32**, D115-119. Available from: http://dx.doi.org/10.1093/nar/gkh131.

**Ashburner, M., Ball, C.A., Blake, J.A., Botstein, D., Butler, H., Cherry, J.M., Davis, A.P., Dolinski, K., Dwight, S.S., Eppig, J.T., Harris, M.A., Hill, D.P., Issel-Tarver, L., Kasarskis, A., Lewis, S., Matese, J.C., Richardson, J.E., Ringwald, M., Rubin, G.M. and Sherlock, G.** (2000) Gene ontology: tool for the unification of biology. The Gene Ontology Consortium. *Nat Genet*, **25**, 25-29. Available from: http://dx.doi.org/10.1038/75556.

**Bao, W., Kojima, K.K. and Kohany, O.** (2015) Repbase Update, a database of repetitive elements in eukaryotic genomes. *Mob DNA*, **6**, 11. Available from: http://dx.doi.org/10.1186/s13100-015-0041-9.

**Bolger, A.M., Lohse, M. and Usadel, B.** (2014) Trimmomatic: a flexible trimmer for Illumina sequence data. *Bioinformatics*, **30**, 2114-2120. Available from: http://dx.doi.org/10.1093/bioinformatics/btu170.

**Borodovsky, M. and McIninch, J.** (1993) GENMARK: Parallel gene recognition for both DNA strands. *Computers & Chemistry*, **17**, 123-133. Available from: http://dx.doi.org/10.1016/0097-8485(93)85004-v.

**Bruna, T., Hoff, K.J., Lomsadze, A., Stanke, M. and Borodovsky, M.** (2021) BRAKER2: automatic eukaryotic genome annotation with GeneMark-EP+ and AUGUSTUS supported by a protein database. *NAR Genom Bioinform*, **3**, lqaa108. Available from: http://dx.doi.org/10.1093/nargab/lqaa108.

**Cantarel, B.L., Korf, I., Robb, S.M., Parra, G., Ross, E., Moore, B., Holt, C., Sanchez, A. and Yandell, M.** (2008) MAKER: an easy-to-use annotation pipeline designed for emerging model organism genomes. *Genome Res*, **18**, 188-196. Available from: http://dx.doi.org/10.1101/gr.6743907.

**Chen, S., Zhou, Y., Chen, Y. and Gu, J.** (2018) fastp: an ultra-fast all-in-one FASTQ preprocessor. *Bioinformatics*, **34**, i884-i890. Available from: http://dx.doi.org/10.1093/bioinformatics/bty560.

**Emms, D.M. and Kelly, S.** (2019) OrthoFinder: phylogenetic orthology inference for comparative genomics. *Genome Biol*, **20**, 238. Available from: http://dx.doi.org/10.1186/s13059-019-1832-y.

**Feng, J., Liu, T., Qin, B., Zhang, Y. and Liu, X.S.** (2012) Identifying ChIP-seq enrichment using MACS. *Nat Protoc*, **7**, 1728-1740. Available from: http://dx.doi.org/10.1038/nprot.2012.101.

**Flynn, J.M., Hubley, R., Goubert, C., Rosen, J., Clark, A.G., Feschotte, C. and Smit, A.F.** (2020) RepeatModeler2 for automated genomic discovery of transposable element families. *Proc Natl Acad Sci U S A*, **117**, 9451-9457. Available from: http://dx.doi.org/10.1073/pnas.1921046117.

**Haas, B.J., Delcher, A.L., Mount, S.M., Wortman, J.R., Smith, R.K., Jr., Hannick, L.I., Maiti, R., Ronning, C.M., Rusch, D.B., Town, C.D., Salzberg, S.L. and White, O.** (2003) Improving the *Arabidopsis* genome annotation using maximal transcript alignment assemblies. *Nucleic Acids Res*, **31**, 5654-5666. Available from: http://dx.doi.org/10.1093/nar/gkg770.

**Haas, B.J., Papanicolaou, A., Yassour, M., Grabherr, M., Blood, P.D., Bowden, J., Couger, M.B., Eccles, D., Li, B., Lieber, M., MacManes, M.D., Ott, M., Orvis, J., Pochet, N., Strozzi, F., Weeks, N., Westerman, R., William, T., Dewey, C.N., Henschel, R., LeDuc, R.D., Friedman, N. and Regev, A.** (2013) De novo transcript sequence reconstruction from RNA-seq using the Trinity platform for reference generation and analysis. *Nat Protoc*, **8**, 1494-1512. Available from: http://dx.doi.org/10.1038/nprot.2013.084.

**Haas, B.J., Salzberg, S.L., Zhu, W., Pertea, M., Allen, J.E., Orvis, J., White, O., Buell, C.R. and Wortman, J.R.** (2008) Automated eukaryotic gene structure annotation using EVidenceModeler and the Program to Assemble Spliced Alignments. *Genome Biol*, **9**, R7. Available from: http://dx.doi.org/10.1186/gb-2008-9-1-r7.

**Hoff, K.J., Lomsadze, A., Borodovsky, M. and Stanke, M.** (2019) Whole-Genome Annotation with BRAKER. *Methods Mol Biol*, **1962**, 65-95. Available from: http://dx.doi.org/10.1007/978-1-4939-9173-0_5.

**Holt, C. and Yandell, M.** (2011) MAKER2: an annotation pipeline and genome-database management tool for second-generation genome projects. *BMC Bioinformatics*, **12**, 491. Available from: http://dx.doi.org/10.1186/1471-2105-12-491.

**Jo, H. and Koh, G.** (2015) Faster single-end alignment generation utilizing multi-thread for BWA. *Biomed Mater Eng*, **26 Suppl 1**, S1791-1796. Available from: http://dx.doi.org/10.3233/BME-151480.

**Kalvari, I., Argasinska, J., Quinones-Olvera, N., Nawrocki, E.P., Rivas, E., Eddy, S.R., Bateman, A., Finn, R.D. and Petrov, A.I.** (2018) Rfam 13.0: shifting to a genome-centric resource for non-coding RNA families. *Nucleic Acids Res*, **46**, D335-D342. Available from: http://dx.doi.org/10.1093/nar/gkx1038.

**Kanehisa, M., Goto, S., Kawashima, S., Okuno, Y. and Hattori, M.** (2004) The KEGG resource for deciphering the genome. *Nucleic Acids Res*, **32**, D277-280. Available from: http://dx.doi.org/10.1093/nar/gkh063.

**Kang, Y.J., Yang, D.C., Kong, L., Hou, M., Meng, Y.Q., Wei, L. and Gao, G.** (2017) CPC2: a fast and accurate coding potential calculator based on sequence intrinsic features. *Nucleic Acids Res*, **45**, W12-W16. Available from: http://dx.doi.org/10.1093/nar/gkx428.

**Kent, W.J.** (2002) BLAT--the BLAST-like alignment tool. *Genome Res*, **12**, 656-664. Available from: http://dx.doi.org/10.1101/gr.229202.

**Kim, D., Paggi, J.M., Park, C., Bennett, C. and Salzberg, S.L.** (2019) Graph-based genome alignment and genotyping with HISAT2 and HISAT-genotype. *Nat Biotechnol*, **37**, 907-915. Available from: http://dx.doi.org/10.1038/s41587-019-0201-4.

**Korf, I.** (2004) Gene finding in novel genomes. *BMC Bioinformatics*, **5**, 59. Available from: http://dx.doi.org/10.1186/1471-2105-5-59.

**Kumar, S., Stecher, G., Li, M., Knyaz, C. and Tamura, K.** (2018) MEGA X: Molecular Evolutionary Genetics Analysis across Computing Platforms. *Mol Biol Evol*, **35**, 1547-1549. Available from: http://dx.doi.org/10.1093/molbev/msy096.

**Langdon, W.B.** (2015) Performance of genetic programming optimised Bowtie2 on genome comparison and analytic testing (GCAT) benchmarks. *BioData Min*, **8**, 1. Available from: http://dx.doi.org/10.1186/s13040-014-0034-0.

**Langfelder, P. and Horvath, S.** (2008) WGCNA: an R package for weighted correlation network analysis. *BMC Bioinformatics*, **9**, 559. Available from: http://dx.doi.org/10.1186/1471-2105-9-559.

**Li, A., Zhou, H., Xiong, S., Li, J., Mallik, S., Fei, R., Liu, Y., Zhou, H., Wang, X., Hei, X. and Wang, L.** (2024a) PLEKv2: predicting lncRNAs and mRNAs based on intrinsic sequence features and the coding-net model. *BMC Genomics*, **25**, 756. Available from: http://dx.doi.org/10.1186/s12864-024-10662-y.

**Li, G., Chen, C., Chen, P., Meyers, B.C. and Xia, R.** (2024b) sRNAminer: A multifunctional toolkit for next-generation sequencing small RNA data mining in plants. *Sci Bull (Beijing)*, **69**, 784-791. Available from: http://dx.doi.org/10.1016/j.scib.2023.12.049.

**Li, H., Handsaker, B., Wysoker, A., Fennell, T., Ruan, J., Homer, N., Marth, G., Abecasis, G., Durbin, R. and Genome Project Data Processing, S.** (2009) The Sequence Alignment/Map format and SAMtools. *Bioinformatics*, **25**, 2078-2079. Available from: http://dx.doi.org/10.1093/bioinformatics/btp352.

**Lowe, T.M. and Eddy, S.R.** (1997) tRNAscan-SE: a program for improved detection of transfer RNA genes in genomic sequence. *Nucleic Acids Res*, **25**, 955-964. Available from: http://dx.doi.org/10.1093/nar/25.5.955.

**Mistry, J., Chuguransky, S., Williams, L., Qureshi, M., Salazar, G.A., Sonnhammer, E.L.L., Tosatto, S.C.E., Paladin, L., Raj, S., Richardson, L.J., Finn, R.D. and Bateman, A.** (2021) Pfam: The protein families database in 2021. *Nucleic Acids Res*, **49**, D412-D419. Available from: http://dx.doi.org/10.1093/nar/gkaa913.

**Myburg, A.A., Grattapaglia, D., Tuskan, G.A., Hellsten, U., Hayes, R.D., Grimwood, J., Jenkins, J., Lindquist, E., Tice, H., Bauer, D., Goodstein, D.M., Dubchak, I., Poliakov, A., Mizrachi, E., Kullan, A.R., Hussey, S.G., Pinard, D., van der Merwe, K., Singh, P., van Jaarsveld, I., Silva-Junior, O.B., Togawa, R.C., Pappas, M.R., Faria, D.A., Sansaloni, C.P., Petroli, C.D., Yang, X., Ranjan, P., Tschaplinski, T.J., Ye, C.Y., Li, T., Sterck, L., Vanneste, K., Murat, F., Soler, M., Clemente, H.S., Saidi, N., Cassan-Wang, H., Dunand, C., Hefer, C.A., Bornberg-Bauer, E., Kersting, A.R., Vining, K., Amarasinghe, V., Ranik, M., Naithani, S., Elser, J., Boyd, A.E., Liston, A., Spatafora, J.W., Dharmwardhana, P., Raja, R., Sullivan, C., Romanel, E., Alves-Ferreira, M., Kulheim, C., Foley, W., Carocha, V., Paiva, J., Kudrna, D., Brommonschenkel, S.H., Pasquali, G., Byrne, M., Rigault, P., Tibbits, J., Spokevicius, A., Jones, R.C., Steane, D.A., Vaillancourt, R.E., Potts, B.M., Joubert, F., Barry, K., Pappas, G.J., Strauss, S.H., Jaiswal, P., Grima-Pettenati, J., Salse, J., Van de Peer, Y., Rokhsar, D.S. and Schmutz, J.** (2014) The genome of *Eucalyptus grandis*. *Nature*, **510**, 356-362. Available from: http://dx.doi.org/10.1038/nature13308.

**Nawrocki, E.P. and Eddy, S.R.** (2013) Infernal 1.1: 100-fold faster RNA homology searches. *Bioinformatics*, **29**, 2933-2935. Available from: http://dx.doi.org/10.1093/bioinformatics/btt509.

**Pertea, M., Pertea, G.M., Antonescu, C.M., Chang, T.C., Mendell, J.T. and Salzberg, S.L.** (2015) StringTie enables improved reconstruction of a transcriptome from RNA-seq reads. *Nat Biotechnol*, **33**, 290-295. Available from: http://dx.doi.org/10.1038/nbt.3122.

**Pruitt, K.D., Tatusova, T. and Maglott, D.R.** (2007) NCBI reference sequences (RefSeq): a curated non-redundant sequence database of genomes, transcripts and proteins. *Nucleic Acids Res*, **35**, D61-65. Available from: http://dx.doi.org/10.1093/nar/gkl842.

**Stanke, M., Keller, O., Gunduz, I., Hayes, A., Waack, S. and Morgenstern, B.** (2006) AUGUSTUS: ab initio prediction of alternative transcripts. *Nucleic Acids Res*, **34**, W435-439. Available from: http://dx.doi.org/10.1093/nar/gkl200.

**Su, G., Morris, J.H., Demchak, B. and Bader, G.D.** (2014) Biological network exploration with Cytoscape 3. *Curr Protoc Bioinformatics*, **47**, 8 13 11-24. Available from: http://dx.doi.org/10.1002/0471250953.bi0813s47.

**Summa, D.S., Malerba, G., Pinto, R., Mori, A., Mijatovic, V. and Tommasi, S.** (2017) GATK hard filtering: tunable parameters to improve variant calling for next generation sequencing targeted gene panel data. *BMC Bioinformatics*, **18**, 119. Available from: http://dx.doi.org/10.1186/s12859-017-1537-8.

**Sun, L., Luo, H., Bu, D., Zhao, G., Yu, K., Zhang, C., Liu, Y., Chen, R. and Zhao, Y.** (2013) Utilizing sequence intrinsic composition to classify protein-coding and long non-coding transcripts. *Nucleic Acids Res*, **41**, e166. Available from: http://dx.doi.org/10.1093/nar/gkt646.

**Thibaud, N.F., DiCuccio, M., Hlavina, W., Kimchi, A., Kitts, P.A., Murphy, T.D., Pruitt, K.D. and Souvorov, A.** (2016) P8008 The NCBI Eukaryotic Genome Annotation Pipeline. *Journal of Animal Science*, **94**, 184-184. Available from: http://dx.doi.org/10.2527/jas2016.94supplement4184x.

**Wang, Y., Tang, H., Debarry, J.D., Tan, X., Li, J., Wang, X., Lee, T.H., Jin, H., Marler, B., Guo, H., Kissinger, J.C. and Paterson, A.H.** (2012) MCScanX: a toolkit for detection and evolutionary analysis of gene synteny and collinearity. *Nucleic Acids Res*, **40**, e49. Available from: http://dx.doi.org/10.1093/nar/gkr1293.
